# Tau‐mediated synaptic dysfunction is coupled with HCN channelopathy

**DOI:** 10.1002/alz.14074

**Published:** 2024-07-12

**Authors:** Despoina Goniotaki, Francesco Tamagnini, Luca Biasetti, Svenja‐Lotta Rumpf, Claire Troakes, Saskia J. Pollack, Shalom Ukwesa, Haoyue Sun, Igor Kraev, Louise C. Serpell, Wendy Noble, Kevin Staras, Diane P. Hanger

**Affiliations:** ^1^ Department of Basic and Clinical Neuroscience Institute of Psychiatry Psychology & Neuroscience Maurice Wohl Clinical Neuroscience Institute King's College London London UK; ^2^ Department of Pharmacy School of Chemistry Food and Pharmacy University of Reading Reading UK; ^3^ Sussex Neuroscience School of Life Sciences University of Sussex Brighton UK; ^4^ Electron Microscopy Suite STEM Faculty The Open University Milton Keynes UK; ^5^ Department of Clinical and Biomedical Sciences University of Exeter Exeter UK

**Keywords:** dementia, hyperpolarization‐activated cyclic nucleotide‐gated channels, neurodegeneration, sag voltage, synapses, tauopathies

## Abstract

**INTRODUCTION:**

In tauopathies, altered tau processing correlates with impairments in synaptic density and function. Changes in hyperpolarization‐activated cyclic nucleotide‐gated (HCN) channels contribute to disease‐associated abnormalities in multiple neurodegenerative diseases.

**METHODS:**

To investigate the link between tau and HCN channels, we performed histological, biochemical, ultrastructural, and functional analyses of hippocampal tissues from Alzheimer's disease (AD), age‐matched controls, Tau35 mice, and/or Tau35 primary hippocampal neurons.

**RESULTS:**

Expression of specific HCN channels is elevated in *post mortem* AD hippocampus. Tau35 mice develop progressive abnormalities including increased phosphorylated tau, enhanced HCN channel expression, decreased dendritic branching, reduced synapse density, and vesicle clustering defects. Tau35 primary neurons show increased HCN channel expression enhanced hyperpolarization‐induced membrane voltage “sag” and changes in the frequency and kinetics of spontaneous excitatory postsynaptic currents.

**DISCUSSION:**

Our findings are consistent with a model in which pathological changes in tauopathies impact HCN channels to drive network‐wide structural and functional synaptic deficits.

**Highlights:**

- Hyperpolarization‐activated cyclic nucleotide‐gated (HCN) channels are functionally linked to the development of tauopathy.
- Expression of specific HCN channels is elevated in the hippocampus in Alzheimer's disease and the Tau35 mouse model of tauopathy.
- Increased expression of HCN channels in Tau35 mice is accompanied by hyperpolarization‐induced membrane voltage “sag” demonstrating a detrimental effect of tau abnormalities on HCN channel function.
- Tau35 expression alters synaptic organization, causing a loosened vesicle clustering phenotype in Tau35 mice.

## 1 BACKGROUND

Synapses are the principal structural and functional units that enable information signaling between neurons. Although synaptic dysfunction is an early correlate of dementia, the pathophysiology of aberrant synaptic signaling remains poorly understood.^1^ In human tauopathies and mouse models of disease, accumulation of phosphorylated and truncated tau, altered tau processing, and aberrant tau localization occur in parallel with reductions in presynaptic protein expression, synapse density, and synaptic function, suggesting a causal role for tau in disease pathogenesis.^2^, ^3^, ^4^

Voltage‐gated hyperpolarization‐activated cyclic nucleotide‐gated (HCN) channels 1 through 4 are a family of non‐selective cation channels that are emerging as central regulators in neuronal signaling.^5^ HCN channels are involved in multiple synaptic processes including the regulation of membrane resistance, the setting of intrinsic membrane excitability, the generation of synaptic potentials, and synaptic vesicle exocytosis.^6^, ^7^, ^8^ Notably, HCN channels selectively associate with regulatory proteins in dendritic spines to drive fine‐tuning of network connectivity.^9^ HCN channel activation generates an inward‐rectifying current (*I*_h_), which reduces dendritic summation and synaptic vesicle release through interaction with calcium channels.^10^, ^11^, ^12^ For example, in the adult entorhinal cortex, increased HCN channel expression dampens spontaneous synaptic vesicle release.^12^ In parallel, the misregulation of HCN channels can result in either gain of function or loss of function in neurological disorders.^13^ HCN channels and the h‐current are implicated in disease progression in transgenic animal models of familial frontotemporal dementia and Alzheimer's disease (AD). Examples of mouse models of dementia in which HCN channels have been implicated include 3xTg mice, 5xFAD mice, J20 mice, ARTE10 mice, and rTg4510 mice, which overexpress mutant forms of *APP*, *MAPT*, and/or *PSEN1* genes.^14^, ^15^, ^16^ These wide‐ranging roles of HCN channels in regulating key structure‐function properties in neurons position them as potentially important substrates to explain the development and progression of tauopathies.

Here we investigate the link between tau abnormalities and HCN channels. We demonstrate an imbalance of HCN channel expression both in *post mortem* AD human hippocampus and in the hippocampus of Tau35 mice, a progressive tauopathy model that exhibits increased tau phosphorylation and deposition of abnormal tau species in the brain, albeit expressing only a minimal amount (≈ 7% of the total tau mRNA) of this human transgene.^17^ Strikingly, selective increases in HCN channels occur as Tau35 mice age and in AD hippocampus. In Tau35 mice, progressive defects in synaptic connectivity and ultrastructure occur alongside functional abnormalities in HCN channel activity and network dynamics, suggesting that pharmacological targeting of specific HCN channels could have significant therapeutic potential in human tauopathy.

## 2 METHODS

### 2.1 Human tissue

#### 2.1.1 Ethics statement

All cases were neuropathologically assessed in accordance with standard criteria after consent for autopsy and research. All studies were conducted under the ethical approval of the King's College London and the Medical Research College (MRC) London Neurodegenerative Diseases Brain Bank.

#### 2.1.2 Preparation of human brain homogenates for western blots

Frozen human hippocampi from AD (modified Braak—Brain Network Europe [BNE] stage VI) and age‐matched controls (BNE stage 0‐I) were obtained from the London Neurodegenerative Diseases Brain Bank (King's College London, UK). Tissue was homogenized in 10 volumes of ice‐cold radio‐immunoprecipitation assay (RIPA) buffer (150 mM NaCl, 1 mM ethylenediaminetetraacetic acid [EDTA], 50 mM Tris‐HCl, 1% [v/v] NP‐40, 0.5% [w/v] sodium deoxycholate, 0.1% [w/v] sodium dodecyl sulfate [SDS]), supplemented with protease inhibitor cocktail (cOmplete, EDTA‐free, Merck Millipore) and phosphatase inhibitor (PhosSTOP, Sigma‐Aldrich) using a glass Dounce homogenizer. Hippocampal homogenates were centrifuged at 10,000 × g for 15 minutes at 4°C and the supernatants were stored at −80°C.

#### 2.1.3 Immunohistochemistry of human brain tissue

Human brain sections (7 µm) were cut from formalin‐fixed paraffin‐embedded tissue blocks and deparaffinized in xylene. Endogenous peroxidase was blocked by immersion in methanol with H_2_O_2_ for 30 minutes and antigen retrieval was carried out using 20 minute microwave heating in citrate buffer, pH 6. After blocking in normal serum, primary antibody (HCN1, HCN2, or HCN3 [rabbit polyclonal]) was applied overnight at 4°C. After two washes in Tris‐buffered saline (TBS), sections were incubated with biotinylated secondary antibody (DAKO), followed by avidin/biotinylated enzyme complex (Vectastain Elite ABC kit, Vector Laboratories). Finally, sections were incubated for 15 minutes with 3,3′‐diaminobenzidine chromogen (0.5 mg/mL; Sigma‐Aldrich) in TBS (pH 7.6) containing 0.05% H_2_O_2_. Sections were counterstained with Harris hematoxylin, and images were acquired using a VS120EL100W, virtual slide microscope (Olympus). Automated image analysis and quantification were performed on antibody‐labeled human hippocampal sections using customized digital analysis apps (Visiopharm A/S).

### 2.2 Mice

#### 2.2.1 Ethics statement

All procedures were conducted in accordance with the Animals (Scientific Procedures) Act, 1986, after approval by the local ethical review committee. All procedures conformed to the Animal Research: Reporting of In Vivo Experiments guidelines 2.0.

Tau35 mice were generated by targeted knock‐in of the Tau35 cDNA construct to the *Hprt* locus, located on the X chromosome, under the control of the human tau promoter as described previously.^17^ In brief, the Tau35 construct encodes a C‐terminal fragment of wild‐type human tau (amino acids 187‐441) with a hemagglutinin (HA) tag fused at the C‐terminus. We assessed male hemizygous transgenic and wild‐type (WT) mice in this study to avoid potential issues of incomplete X chromosome inactivation in female mice.

#### 2.2.2 Preparation of mouse brain homogenates for western blots

Mice were sacrificed by cervical dislocation. Brains were removed and snap frozen in dry ice and stored at −80°C. Tissue was lysed by ultrasonication (sonication parameters: amplitude 40%; pulse 4 seconds; time: 30 seconds) using a Vibra‐Cell ultrasonic liquid processor (Model no. VCX 130, Sonics and Materials) in ice‐cold RIPA buffer (150 mM NaCl, 1 mM EDTA, 50 mM Tris‐HCl, 1% [v/v] NP‐40, 0.5% [w/v] sodium deoxycholate, 0.1% [w/v] SDS), supplemented with protease inhibitor cocktail (cOmplete, EDTA‐free, Merck Millipore) and phosphatase inhibitor (PhosSTOP, Sigma‐Aldrich), followed by incubation on ice for 3 minutes, repeating this cycle three times. Lysed tissue was centrifuged at 10,000 × g for 15 minutes at 4°C and the supernatants were stored at −80°C.

**RESEARCH IN CONTEXT**

1. **Systematic review**: The authors used PubMed to review existing literature on potential relationships between hyperpolarization‐activated cyclic nucleotide‐gated (HCN) channels and neurodegeneration. HCNs are emerging as central regulators in neuronal signaling but to date, tauopathy has not been linked to HCN channel dysfunction.
2. **Interpretation**: Our findings have uncovered an integrating hypothesis, in which we propose upregulation of specific HCN channels as a potential mechanism involved in the development of neurodegenerative disease that may contribute to the selective vulnerability of hippocampal neurons in tauopathies.
3. **Future directions**: This study was limited to understanding the role of HCN channels in Alzheimer's disease. Future studies could expand this work to include an understanding of (1) the potential role of specific HCN channels in other human tauopathies, (2) the impact of increased HCN channel expression on function in specific neural cell types, (3) the effects of targeting specific HCN channels with clinically available drugs to determine their therapeutic potential in human tauopathies.

#### 2.2.3 Preparation of mouse brain tissue for immunolabeling and Golgi–Cox staining

Mice were sacrificed using terminal anesthesia, perfused with phosphate‐buffered saline (PBS), and post‐fixed in 4% (w/v) paraformaldehyde (PFA) overnight at 4°C. Brain sections (50‐200 µm) were prepared using a VT1000 S Vibrating blade microtome (Leica Biosystems) and stored free‐floating in cryoprotectant (30% [v/v] ethylene glycol, 15% [w/v] sucrose in PBS) at −20°C.

For immunofluorescence, 50 µm sections were washed in PBS, blocked in 3% (v/v) goat serum (Sigma‐Aldrich), in 0.1% (v/v) Triton X‐100 (ThermoFisher Scientific) in PBS, and incubated in primary antibody in PBS overnight at 4°C. After washing, sections were incubated in the appropriate fluorophore‐conjugated secondary antibody for 4 to 6 hours at 4°C, and counterstained with 4′,6‐diamidino‐2‐phenylindole (DAPI), before mounting in fluorescence mounting medium (S3023, Agilent Dako). Whole brain sections were imaged using a VS120EL100W, virtual slide microscope (Olympus) equipped with ×20, 0.75 NA and ×40, 0.95 NA, air objectives. Automated image analysis and quantification were performed on antibody‐labeled mouse brain sections using customized digital analysis apps (Visiopharm A/S). High‐magnification images of neurons in the CA1 and CA3 regions of the hippocampus were collected using the VT‐iSIM microscope (VisiTech International) with a Hamamatsu Flash 4.0 sCMOS camera.

For Golgi–Cox staining, 200 µm sections^18^ were stained according to the manufacturer's instructions (sliceGolgi Kit, Bioenno Lifesciences) and imaged using a Nikon Ti‐E two‐camera microscope (×60, 1.4 NA, oil objective). Three‐dimensional digital reconstruction of dendritic, axonal, and spine structures was performed using Neurolucida software (BFM Bioscience). Whole brain sections were imaged using a VS120EL100W virtual slide microscope (Olympus) equipped with ×20, 0.75 NA and ×40, 0.95 NA, air objectives.

#### 2.2.4 Preparation of mouse brain for ultrastructural analysis

Mice were sacrificed using terminal anesthesia, perfused with PBS, and post‐fixed in 4% (w/v) PFA, and 0.1% (w/v) glutaraldehyde in PBS, overnight at 4°C. Brains were removed and 300 µm transverse brain sections were prepared using a vibratome (VT1000 S vibrating blade microtome, Leica Biosystems). Fixative was replaced with 0.2 M sodium cacodylate and 0.2 M sodium cacodylate, supplemented with 0.02 M CaCl_2_. Sections were treated with 2% (w/v) osmium tetroxide for 1 hour on ice. After washing in ultra‐pure water, sections were stained for 20 minutes in 0.1% (w/v) thiocarbohydrazide at ambient temperature, followed by three washes in water. Sections were treated with 2% (w/v) osmium tetroxide in 0.2 M sodium cacodylate, supplemented with 0.02 M CaCl_2_ and 1.5% (w/v) potassium ferrocyanide. After a further water wash, sections were stained by incubating in 1% (w/v) uranyl acetate overnight at 4°C. Stained sections were dehydrated in ascending concentrations of ethanol, then in acetone, before flat embedding in ascending concentrations of Durcupan resin (25% [v/v] for 2 hours, 50% [v/v] for 2 hours, 70% [v/v] for 2 hours, 100% [v/v] overnight). The fresh resin was added and polymerized using a ultraviolet (UV) light translinker for 3 to 4 days at 4°C (UVP TL‐2000 Translinker). The stained and embedded hippocampus was dissected, glued onto a BEEM capsule, mounted in an ultramicrotome (Leica), and ultrathin 70 nm sections were collected on hexagonal 300‐mesh nickel grids (3.05 mm; Agar Scientific).

#### 2.2.5 Western blots

Protein concentration was determined using a bicinchoninic acid protein assay, according to the manufacturer's instructions (Pierce BCA Protein Assay Kit, ThermoFisher Scientific). Samples in Laemmli sample buffer were incubated at 95°C for 10 minutes and electrophoresed on 10% (w/v) SDS‐polyacrylamide gels. Separated proteins were transferred to nitrocellulose membranes, blocked in Intercept (TBS) Blocking Buffer (LI‐COR Biosciences), and incubated in primary antibodies, overnight at 4°C. After washing in TBS containing 0.02% (v/v) Tween 20, membranes were incubated with appropriate fluorophore‐conjugated secondary antibodies for antigen detection and imaged (Odyssey imager, LI‐COR Biosciences). ImageStudio Lite software (LI‐COR Biosciences) was used for the quantification of western blots.

#### 2.2.6 Primary neuron culture

Primary hippocampal and cortical neurons were prepared from embryonic (E) day 16.5 to 18.5 Tau35 and WT mice^17^ and cultured as described previously.^19^ Hippocampal neurons were transfected at 4 days in vitro (DIV) using Lipofectamine 2000 (ThermoFisher Scientific) and pEGFP‐C1 plasmid (Addgene, https://media.addgene.org/data/plasmids/58/58473/58473‐map_X0HCgs9WaXq6.pdf) to exogenously express enhanced green fluorescent protein (eGFP). Transfection efficiency was ≈ 15%. Neurons were then fixed at 6, 9, and 14 DIV to monitor dendritic branching and quantify spine density. Untransfected neurons were cultured for 6, 9, and 14 DIV and either fixed in 4% (w/v) PFA for 15 minutes and labeled with antibodies for immunofluorescence or lysed for analysis on western blots. For immunofluorescence staining, non‐specific sites were blocked for 30 minutes in 3% (v/v) goat serum (Sigma‐Aldrich), in 0.1% (v/v) Triton X‐100 (ThermoFisher Scientific) in PBS, and cells were further incubated with primary antibodies overnight at 4°C, followed by incubation with Alexa‐conjugated secondary antibodies. Before mounting (fluorescence mounting medium, S3023, Agilent Dako), neurons were counterstained with 300 nM DAPI in PBS (Sigma‐Aldrich). For blocking peptide experiments, 10 times excess of blocking peptide was incubated with antibody overnight at 4°C.

#### 2.2.7 Negative‐stain transmission electron microscopy

Transmission electron microscopy projection images were collected using a JEM1400‐Plus microscope (JEOL) operated at 100 kV and equipped with a Gatan OneView camera (4k 9 4k). High magnification (20,000×) and panoramic (5000×) micrographs were obtained for synaptic vesicle and synapse density analyses, respectively. Only synapses with defined postsynaptic densities and containing between 10 and 250 synaptic vesicles (SVs), were included in the ultrastructural analysis. SVs and synapses were manually counted in coded images. Synaptic vesicle diameter and presynaptic area were measured using ImageJ.^20^ For three‐dimensional reconstructions, serial micrographs were aligned using Reconstruct software (https://synapseweb.clm.utexas.edu/software‐0). Nearest neighbor analysis was performed by marking vesicle coordinates and comparing the linear distances between their centers using custom‐written MATLAB scripts.^21^ Cumulative frequency plots for each synapse per condition were averaged to allow comparisons between WT and Tau35 mice at different age points.

#### 2.2.8 Immunoelectron microscopy

Mice were perfused with a fixative solution (4% [w/v] PFA, 0.1% [w/v] glutaraldehyde in PBS) and brain tissue was isolated. Samples of 1 mm^3^ containing hippocampus were excised into fixative solution and stored at 4°C overnight. Samples were washed by rotating in PBS for 4 hours at 4°C, and then 300 µm sections were cut using a Leica VT1000 S vibrating blade microtome (Leica Biosystems). Sections were dehydrated in increasing ethanol concentrations (30%, 50%, 75%, 90%, and 100%, each for 20 minutes). Samples were prepared for embedding in a 2:1 ratio of ethanol:resin (UNICRYL; BBI Solutions) for 2 hours, followed by a secondary incubation in a 1:2 ratio of ethanol:resin for 30 minutes. After a final incubation in 100% resin overnight at 4°C, fresh resin was applied, and the sample was transferred to a BEEM capsule (Agar Scientific) for resin polymerization under UV light for 3 to 4 days at 4°C.

Polymerized tissue blocks were mounted on the sample holder of a Leica Ultracut UCT ultramicrotome. Ultrathin sections (100 nm) of embedded mouse hippocampus were cut using a diamond knife (Ultra Diamond Knife −45°, 3 mm) on an ultramicrotome (Leica Ultracut UCT). Sections were collected on square 400 mesh nickel grids (3.05 mm; Athene type 9, Agar Scientific) prior to immunogold labeling.^22^ Grids were incubated with 10% (v/v) normal goat serum in PBS+ (PBS containing 1% [w/v] bovine serum albumin, 0.05% [v/v] Tween‐20, 10 mM EDTA, 0.02% sodium azide, pH 8.2) for 1 hour at ambient temperature. Sections were labeled with HCN1 (guinea pig polyclonal) or HCN3 (rabbit polyclonal) antibodies diluted 1/50 in PBS+, and incubated overnight at 4°C. The sections were then immunolabeled with 10 nm gold‐labeled goat secondary antibodies (anti‐guinea pig immunoglobulin G [IgG; ab39617] or anti‐rabbit IgG [ab39601], Abcam) diluted 1/10 in PBS+, for 1 hour at ambient temperature. After washing in PBS+ and distilled water, grids were post‐fixed in 2.5% (w/v) glutaraldehyde and post‐stained in 0.5% (w/v) uranyl acetate (0.22 µm‐filtered) for 1 hour, followed by a 3% (w/v) lead citrate solution for 1 hour. The washed grids were dried and transmission electron microscopy projection images were collected using a JEOL JEM1400 transmission electron microscope operated at 80 kV using an AMT XR60 digital camera.

#### 2.2.9 Patch‐clamp recording of cultured hippocampal and cortical neurons

Neurons were cultured on 13 mm coverslips as previously described.^19^ Immediately prior to recording, coverslips were transferred to a submerged‐style recording chamber where they were continuously perfused (1 to 3 mL/min) with Carbogen (95% O_2_, 5% CO_2_), artificial cerebrospinal fluid (aCSF, 124 mM NaCl, 3 mM KCl, 24 mM NaHCO_3_, 2 mM CaCl_2_, 1.25 mM NaH_2_PO_4_, 1 mM MgSO_4_, 10 mM D‐glucose) maintained at 34°C with a temperature control system (Scientifica). Neurons were visualized with an infrared, differential interference contrast microscope (Scientifica). Borosilicate glass micropipettes were pulled with a horizontal puller (Sutter Instrument) to an access resistance of 5 to 9 MΩ. Single micropipettes were filled with intracellular solution (120 mM K‐gluconate, 10 mM Na_2_‐phosphocreatine, 0.3 mM Na_2_‐GTP, 10 mM HEPES, 4 mM KCl, 4 mM Mg‐ATP, pH 7.2, 280‐290 mOsm). Some recordings were conducted in the presence of 30 µM Alexa Fluor™ 488 dye in the intracellular solution. After the clamping of a stable whole‐cell configuration, the junction potential *V*_j_ = 15 mV arose due to the difference in composition between intracellular and extracellular solutions; this was corrected arithmetically during analysis. All signals were amplified with a Multiclamp 700B amplifier and digitized using a Digidata 1550B. For whole‐cell recordings, a hyperpolarizing current of 500 ms, −100 pA was injected, and the consequent plasma membrane voltage (*V*_m_) deflection was measured. To avoid the possibility of bias arising from cell‐to‐cell variability of the resting membrane potential, all recordings were conducted in the presence of a constant current, holding the pre‐stimulus at *V*_m _= −80 mV. For voltage‐clamp recording of spontaneous excitatory postsynaptic currents (sEPSCs), a gap‐free protocol at a holding potential *V*_h _= −70 mV was used. Data analysis for quantifying the frequency and the waveform properties of sEPSCs used Clampfit (Molecular Devices). The template search analysis tool embedded in Clampfit was used to identify single sEPSCs for each cell, with a manual check to identify any false positives and negatives. The average frequency was calculated as the number of events detected during 30 seconds, expressed in Hz. Averaged sEPSCs from all detected events were used to measure sEPSC waveform properties in each cell. Analysis tools in Clampfit were used to quantify the sEPSC rate of rise, peak, and halfwidth. Current‐voltage (I‐V) curves for voltage‐gated, non‐inactivating, outward currents were carried out as follows. Series resistance was compensated for (10%‐95% correction) and the capacitance of the pipette was neutralized. Outward, non‐inactivating voltage‐gated currents were evoked by applying *n* = 12, 30 ms, 10 mV voltage steps, starting from an initial V_h_ of −90 mV. Each recorded current was leak subtracted and normalized to membrane capacitance to measure the specific current. The specific conductance (*G*) was calculated as the ratio between the specific current and the electrochemical force for potassium (*E*_K_ = 100 mV). Cell‐to‐cell Boltzmann sigmoidal fit was used to estimate the maximal specific conductance (*G*_max_) and the half‐activation voltage (*V*_½_) for each neuron. Current clamp recordings used a set pre‐stimulus *V*_m_ of −80 mV, with a constant current injection (apart from recording the resting membrane potential). Passive electrical properties were measured by injecting a −100 pA, 500 ms square current step. The voltage deflection caused by this current injection followed a single exponential decay function. The steady state voltage deflection (A) and the voltage extrapolated ad infinitum (B) for a single exponential function fit between 10% and 90% of the minimum point (C) of the voltage deflection, were used to calculate cell input resistance (R_in_) and sag. Sag_sub_ was calculated as (C – A)/C and sag_fit_ was calculated as (B – A)/B, expressed as a percentage. Sag was measured as subtraction of the negative peak (sag_sub_) and of the extrapolated first exponential fit of the *V*_m_ decay (sag_fit_) upon injection of a hyperpolarizing step.^23^

### 2.3 Antibodies

The following primary antibodies were used in this study: mouse PHF‐1 (generously provided by Prof Peter Davies, western blot, 1/2000, RRID:AB_2315150), rabbit anti‐Tau (Agilent Dako, western blot, 1/5000, RRID:AB_10013724), mouse anti‐NSE (ThermoFisher Scientific, western blot, 1/5000, RRID:AB_560397), mouse anti‐GAPDH (Santa Cruz, western blot, 1/5000, RRID:AB_627679), mouse anti‐synapsin 1 (EMD Millipore, western blot, 1/1000, cat. number: MABN894), mouse anti‐synaptotagmin (BD Biosciences, western blot, 1/1000, RRID:AB_397810), rabbit anti‐postsynaptic density protein‐95 (Cell Signaling, western blot, 1/2000, RRID:AB_1264242), rabbit anti‐HCN1 (Alomone Labs, immunostaining, 1/100, RRID:AB_2039900), guinea pig anti‐HCN1 (Alomone Labs, immunostaining, 1/50, RRID:AB_2756625), rabbit anti‐HCN1 (Novus Biologicals, western blot, 1/1000, NBP2‐14084), mouse anti‐HCN2 (Novus Biologicals, western blot, 1/1000, NBP2‐12895), rabbit anti‐HCN2 (Alomone Labs, immunostaining, 1/100, RRID:AB_2313726), rabbit anti‐HCN3 (Alomone Labs, immunostaining, 1/100, RRID:AB_2039904), rabbit anti‐HCN3 (Alomone Labs, western blot, 1/1000, RRID:AB_2039904), rabbit anti‐HCN3 (Alomone Labs, immunostaining, 1/50, AB_2756742), guinea pig anti‐HCN4 (Alomone Labs, immunostaining, 1/50, RRID:AB_2340957).

For immunolabeling, fluorescently labeled highly cross‐absorbed secondary antibodies were purchased from either ThermoFisher Scientific or Jackson ImmunoResearch and used at 1/1000 dilution. For immunogold labeling, immunogold reagents with a gold particle diameter of 10 nm were purchased from Abcam and used at 1/10 dilution. For western blots secondary antibodies were purchased from LI‐COR Biosciences and used at 1/10,000 dilution.

### 2.4 Quantification and statistical analysis

For all mouse experiments, experimental or genetically matched controls were used and tested in the same session. For western blot analysis, Studio Lite software (LI‐COR Biosciences) was used for quantification. For human hippocampal and whole mouse brain sections, images were acquired using a VS120EL100W virtual slide microscope (Olympus)×20, 0.75 NA,. Automated image analysis and quantification were performed using customized digital analysis apps (Visiopharm A/S). High magnification images of neurons in the CA1 and CA3 regions of the hippocampus were collected using the Vt‐iSIM microscope with Hamamatsu Flash 4.0 sCMOS camera. Golgi–Cox stained sections were imaged using a Nikon Ti‐E two‐camera microscope (×60, 1.4 NA, oil objective). Three‐dimensional digital reconstruction and quantification of dendritic, axonal, and spine structures were performed using Neurolucida software (BFM Bioscience). Transmission electron microscopy projection images were collected using a JEM1400‐Plus microscope (JEOL) operated at 100 kV and equipped with a Gatan OneView camera (4k 9 4k). High magnification (20,000×) and panoramic (5000×) micrographs were obtained for synaptic vesicle and synapse density analyses, respectively. Only synapses with defined postsynaptic densities and containing between 10 and 200 SVs, were included in the ultrastructural analysis. SVs and synapses were manually counted in blinded coded images to avoid bias. Synaptic vesicle numbers, diameter, and presynaptic area as well as synapse density were measured using ImageJ. For electrophysiological recordings, analysis tools in Clampfit (Molecular Devices) were used to quantify the frequency and the waveform properties of sEPSCs as well as the sEPSC rate of rise, peak, and halfwidth.

For all datasets the statistical significance was assessed as follows: for two‐group comparisons, two‐tailed unpaired Student *t* tests were used to estimate statistical significance between means; for three or more group comparisons, two‐way analysis of variance (ANOVA) followed by Tukey's post hoc test, was used to estimate statistical significance between means. In all cases, statistical analyses were performed using Prism (GraphPad, version 10). The sample number, number of experiments, and statistical information are stated in the corresponding figure legends. In the figures, asterisks denote statistical significance as follows: ^*^ *P* < 0.05, ^**^ *P* < 0.01, ^***^ *P* < 0.001, ^****^ *P* < 0.0001. Error bars represent the standard error of the mean (SEM).

## 3 RESULTS

### 3.1 Increased tau phosphorylation and HCN channel expression in human *post mortem* AD brain and in Tau35 mice

**FIGURE 1:**
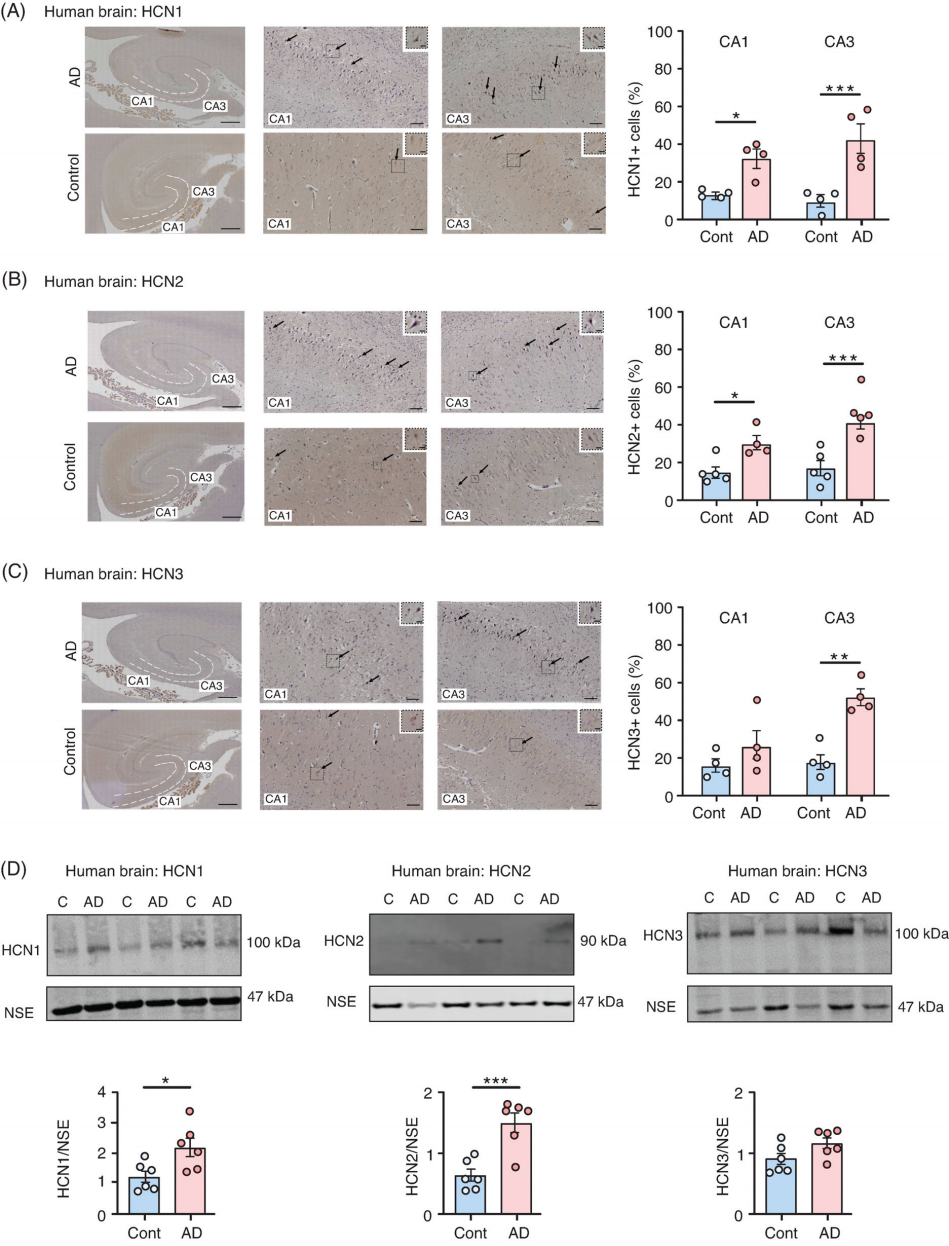
Increased HCN1, HCN2, and HCN3 channel expression in human *post mortem* AD brain. A‐C, HCN1, HCN2, and HCN3 channel immunolabeling (arrows) in the hippocampus (CA1 and CA3 regions) of *post mortem* AD and age‐matched control (Cont) brain. Scale bars: left panels 1000 µm; center and right panels 100 µm; insets show higher magnification of indicated areas, scale bars: 20 µm. Quantification of the percentage of HCN1^+^, HCN2^+^, and HCN3^+^ cells in the CA1 and CA3 regions is shown as mean ± SEM, *n* = 4 brains per group. Student *t* test, ^*^ *P* < 0.05, ^**^ *P* < 0.01, ^***^ *P* < 0.001. D, Western blots of AD and control hippocampus were probed with antibodies to HCN1, HCN2, HCN3, and NSE. Quantification of the blots is shown as mean ± SEM, *n* = 6 brains per group. Student *t* test, ^*^ *P* < 0.05, ^**^ *P* < 0.01, ^***^ *P* < 0.001. AD, Alzheimer's disease; CA, cornu ammonis; HCN, hyperpolarization‐activated cyclic nucleotide‐gated; NSE, neuron‐specific enolase; SEM, standard error of the mean.

To examine a possible role for HCN channels in neurodegenerative pathology, we performed histological and biochemical analyses on hippocampal tissues from *post mortem* AD and age‐matched control human brains. We focused our investigation on the HCN1, 2, and 3 isoforms because these are known to be expressed in the human hippocampus, whereas HCN4 is only very weakly expressed, if at all.^24^ Immunohistochemistry showed elevated HCN channels in the cornu ammonis (CA) 1 and CA3 hippocampal regions in the AD brain, with statistically significant increases in the percentages of HCN1^+^, HCN2^+^, and HCN3^+^ cells in CA3, and in HCN1^+^ and HCN2^+^ cells in the CA1 region (Figure 1A‐C). Analysis of AD and control brain homogenates on western blots verified the increased HCN1 and HCN2 expression in the AD hippocampus, showed no change in expression of HCN3 (Figure 1D); no expression of HCN4 was detected. Other synaptic markers, including synapsin‐1, synaptotagmin, and postsynaptic density protein‐95, did not show significant differences between AD and the control brain (Figure S1A in supporting information). As expected, both phosphorylated tau (PHF‐1 epitope, Ser396/Ser404) and total tau were increased in AD compared to the control brain (Figure S1B).

Next, we examined the expression of HCN channels and synaptic markers in the brains of Tau35 mice, a model of progressive tauopathy that shows increased tau phosphorylation and deposition of abnormal tau species in parallel with deficits in cognitive and motor function, and reduced lifespan.^17^ Changes in HCN channels and synaptic markers as Tau35 mice age and disease develops, could have predictive value for determining whether network‐wide structural and functional synaptic alterations are likely to progress and induce pathological alterations.

**FIGURE 2:**
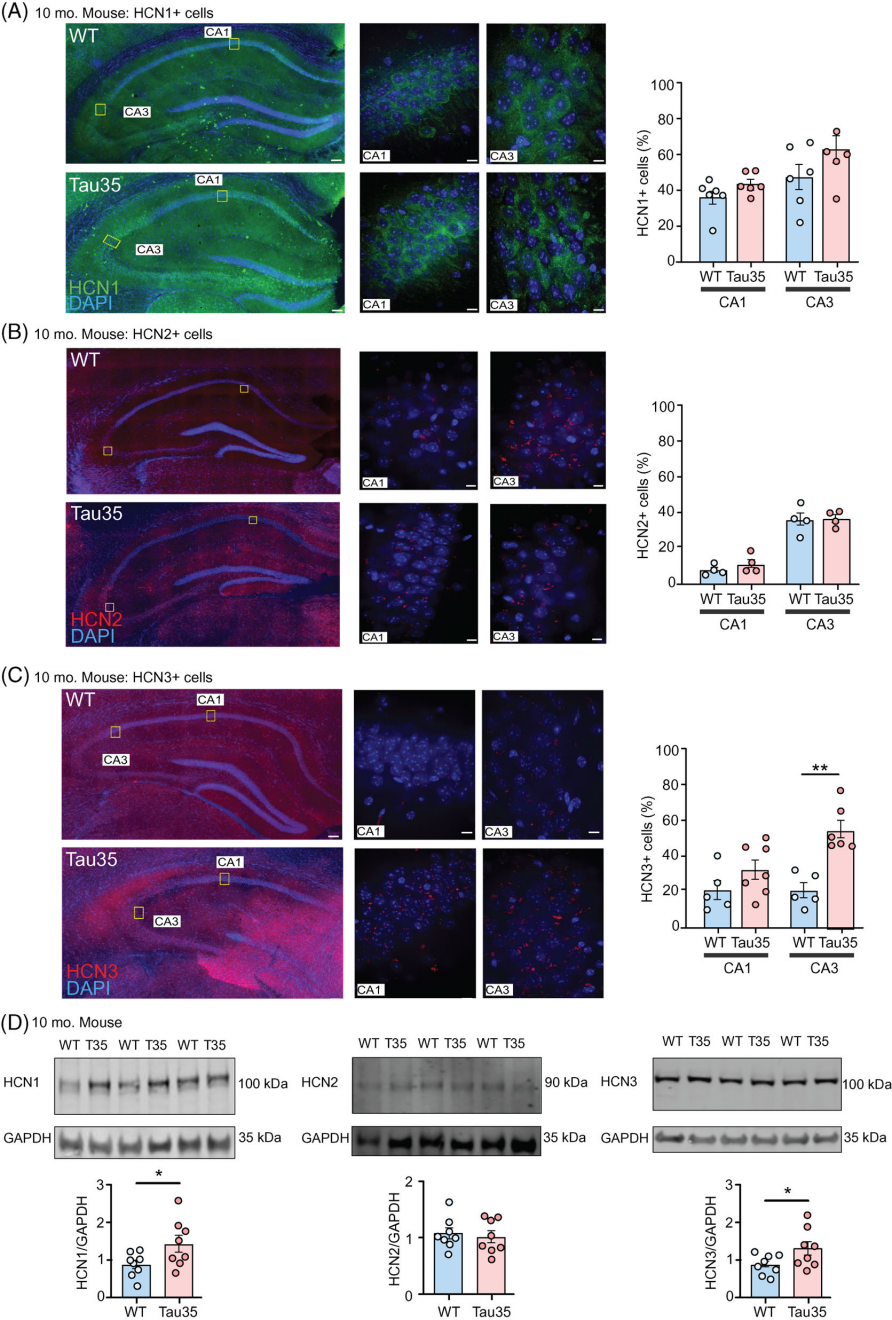
Increased HCN channel expression and tau phosphorylation in postsymptomatic Tau35 mouse hippocampus. Immunofluorescence labeling of (A) HCN1, (B) HCN2, and (C) HCN3 in brain sections from WT and Tau35 postsymptomatic mice (aged 10 months). Scale bars: 100 µm (left panels) and 10 µm (right panels). The percentage of HCN^+^ cells in the CA1 and CA3 regions is shown in the graphs as mean ± SEM, *n* = 4‐7 brains per group. Student *t* test, ^**^ *P* < 0.01. D, E, Western blots of hippocampal homogenates from WT and Tau35 mice aged 10 months, were probed with antibodies to HCN1, HCN2, HCN3, and GAPDH. Quantification of the blots is shown in the graphs as mean ± SEM, *n* = 8 brains per group. Student *t* test, ^*^ *P* < 0.05, ^**^ *P* < 0.01. CA, cornu ammonis; GAPDH, glyceraldehyde 3‐phosphate dehydrogenase; HCN, hyperpolarization‐activated cyclic nucleotide‐gated; SEM, standard error of the mean; WT, wild type.

We first performed histological and biochemical analyses on the hippocampus from postsymptomatic Tau35 (10 months old) mice, when overt hippocampal tau pathology is apparent,^17^ and WT mouse brain, using antibodies to HCN1, HCN2, and HCN3 (Figure 2). We found statistically significant increases in the percentage of HCN3^+^, but not HCN1^+^ and HCN2^+^, cells in the CA3 region of the hippocampus in Tau35 mice (Figure 2A‐C). Analysis of Tau35 and WT hippocampal tissue on western blots showed significant increases in expression of both HCN1 and HCN3, but not HCN2 channels (Figure 2D), as well as increased tau phosphorylation in Tau35 mice (Figure S2A in supporting information). In parallel, western blots of the hippocampus of presymptomatic mice (4 months old) were probed with antibodies to HCN1, HCN2, and HCN3 to monitor whether changes in the expression of HCN channels occur before overt cognitive decline in Tau35 mice. Quantification of the blots showed significant increases in the expression of HCN1, HCN2, and HCN3 channels together with increased phosphorylated tau in young Tau35 mice (Figure S2B, C). Marked decreases in the presynaptic marker synapsin‐1 were observed in Tau35 mice at both ages, whereas synaptotagmin and postsynaptic density protein‐95 were unchanged (Figure S2D, E). Although synapsin‐1 shows a reduction in both Tau35 mice and AD brain, the differences were not significant in the case of the latter (Figure S1A), presumably reflecting the small number of human samples we had available to analyze or subtle differences in the strength of the phenotype. Because synapsin‐1 is essential for the clustering of SVs at the active zone,^25^ the reduction in synapsin‐1 in Tau35 mice could potentially affect SV organization and distribution at synapses.

### 3.2 Reduced dendritic branching and spine density in Tau35 mouse brain and primary hippocampal neurons

**FIGURE 3:**
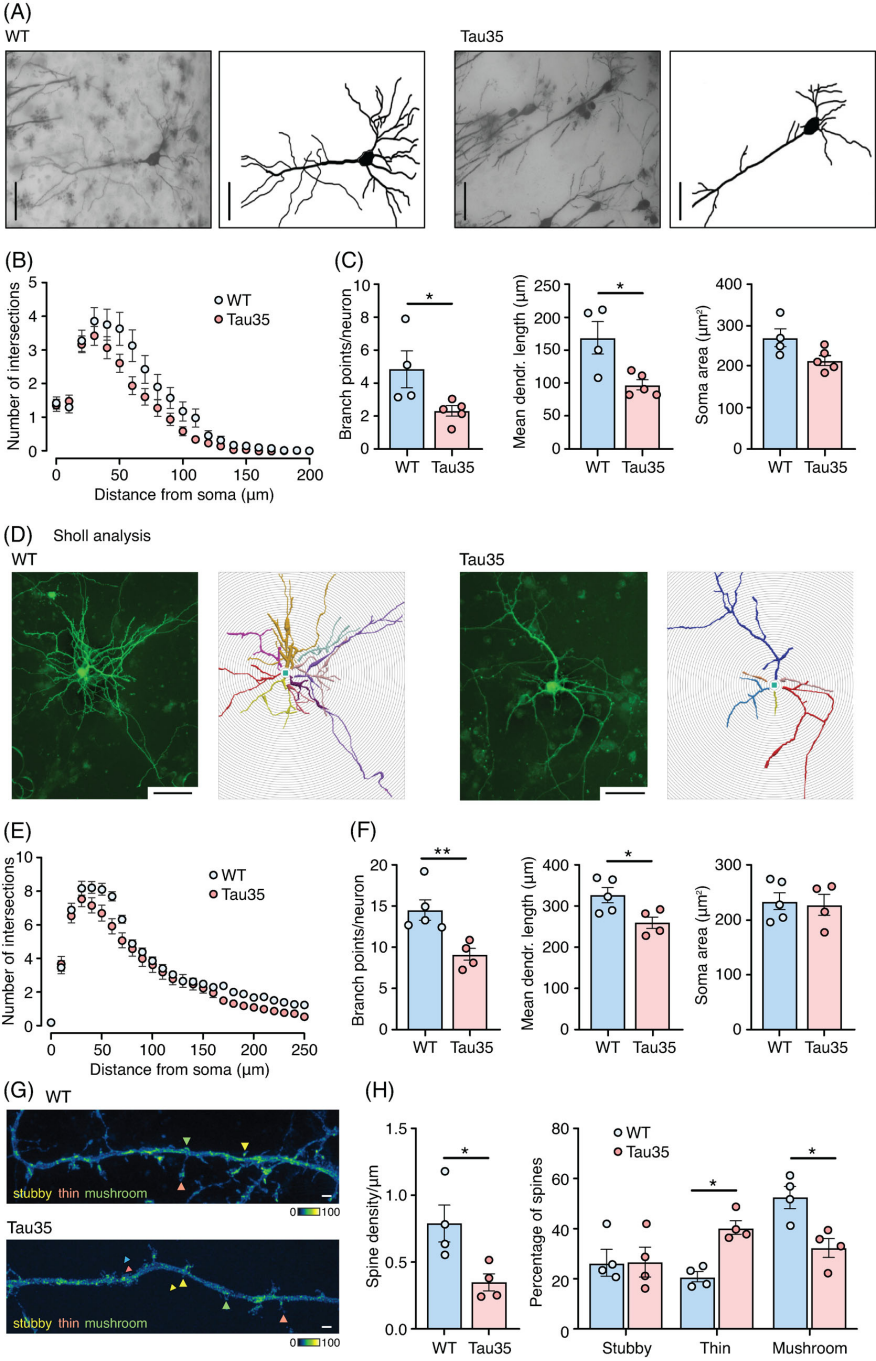
Progressive loss of dendritic complexity and spine density in Tau35 mouse brain and primary hippocampal neurons. A, Golgi–Cox stained WT and Tau35 CA1 hippocampal neurons in 10‐month‐old mice. Scale bars: 50 µm. B, Sholl analysis demonstrates the reduced complexity of basal dendrites in Tau35 mice. The graph shows quantification of the mean ± SEM, *n* = 40 neurons from 4 to 5 mice of each genotype. Two‐way ANOVA, *P* < 0.001. C, Graphs show the number of primary branch points per neuron, mean dendrite length, and soma area in CA1 neurons of WT and Tau35 mice, *n* = 40 neurons from 4 to 5 mice of each genotype. Student *t* test, ^*^ *P* < 0.05. D, Fluorescence images (left) and Neurolucida drawings (right) of WT and Tau35 primary hippocampal neurons, transfected at 4 DIV with a plasmid expressing eGFP and imaged at 14 DIV. Scale bars: 50 µm. E, Sholl analysis of dendritic branching in WT and Tau35 hippocampal neurons at 14 DIV shows reduced dendritic complexity in Tau35 neurons. The graph shows quantification of the mean ± SEM, *n* = 52 neurons of each genotype from 4 to 5 independent experiments. Two‐way ANOVA, *P* < 0.001. F, Graphs show the number of dendritic branch points, mean dendrite length, and soma area in WT and Tau35 hippocampal neurons at 14 DIV, *n* = 52 neurons of each genotype, from 4 to 5 independent experiments. Student *t* test, ^*^ *P* < 0.05, ^**^ *P* < 0.01. G, Representative images of dendrites from WT and Tau35 hippocampal neurons transfected with a plasmid expressing eGFP and imaged at 14 DIV. Stubby spines (yellow), thin spines (orange), and mushroom spines (green) are indicated. Scale bars: 1 µm. H, Graphs show quantifications of spine density (number of spines per µm dendrite length) and percentage of each spine type in WT and Tau35 hippocampal neurons at 14 DIV; *n* = 50 neurons of each genotype from 4 independent experiments. Student *t* test, ^*^ *P* < 0.05. ANOVA, analysis of variance; CA, cornu ammonis; DIV, days in vitro; eGFP, enhanced green fluorescent protein; SEM, standard error of the mean; WT, wild type.

To look for morphological correlates of functional deficits in Tau35 neurons, we conducted Sholl analysis in Golgi–Cox stained hippocampus (CA1 region) in 10‐month‐old Tau35 and WT mice (Figure 3A, B). We found significant reductions in the number of dendritic branch points and mean dendrite length, but not in the soma area of Tau35 neurons compared to WT mice (Figure 3C). Notably, expression of Tau35 significantly decreased overall arborization of basal dendrites of hippocampal neurons (two‐way ANOVA, *P* < 0.0001), a possible correlate of reduced synaptic strength. In contrast, a similar analysis of basal dendrites in 4‐month‐old mice, when Tau35 mice exhibit minimal tau pathology, did not reveal any significant differences in the number of dendritic branch points, mean dendrite length, or soma area (Figure S3A, B in supporting information). No statistically significant changes were observed in apical dendrites (data not shown). Our results show that Tau35 expression reduces dendritic arborization and this loss of neuronal connectivity parallels the appearance of tau pathology and cognitive decline in Tau35 mice.^17^

We established an in vitro model of Tau35 neurons and conducted a Sholl analysis in eGFP‐expressing hippocampal neurons, which were transfected at 4 DIV and imaged at 6, 9, and 14 DIV. In 6 DIV neurons, we observed no effect of Tau35 on dendritic branching and mean dendrite length (Figure S3C), while there was a 14% reduction in branching by 9 DIV (Figure S3D, two‐way ANOVA, *P* < 0.01). However, there was a striking reduction in the dendritic complexity of Tau35 neurons at 14 DIV (Figure 3D, E, two‐way ANOVA, *P* < 0.001), paralleled by an increase in tau phosphorylation at the PHF‐1 epitope (Figure S3E), suggesting a relationship between arborization of hippocampal neurons and tau phosphorylation. Specifically, the number of dendritic branch points in Tau35 neurons decreased by 44% and the mean dendrite length reduced by 20% compared to WT neurons, without any change in the soma area (Figure 3F). Correct formation of the dendritic arbor has important consequences for synaptic function in neurons. As such, this marked reduction in dendritic complexity suggests that the presence of Tau35 could result in decreased synaptic activity and/or synaptic strength.

Next, to provide further insights into functional changes, we characterized dendritic spine morphology, given that both spine number and structure are known correlates of synaptic efficacy.^26^, ^27^ The number of each morphologically defined dendritic spine type was determined from three‐dimensional reconstructions of eGFP‐expressing hippocampal neurons at 14 DIV (Figure 3G). Dendritic spine density was reduced by 59% (*P* < 0.05) in Tau35 neurons (Figure 3H). Moreover, Tau35 expression specifically reduced the proportion of mushroom spines and increased the number of immature thin spines (Figure 3H, *P* < 0.05). The decrease in mushroom spine number suggests that Tau35 expression has a potentially negative impact on synaptic transmission and could limit synaptic plasticity^28^ and enhance synaptotoxicity.

### 3.3 Ultrastructural changes in hippocampal presynaptic terminals of Tau35 mice

**FIGURE 4:**
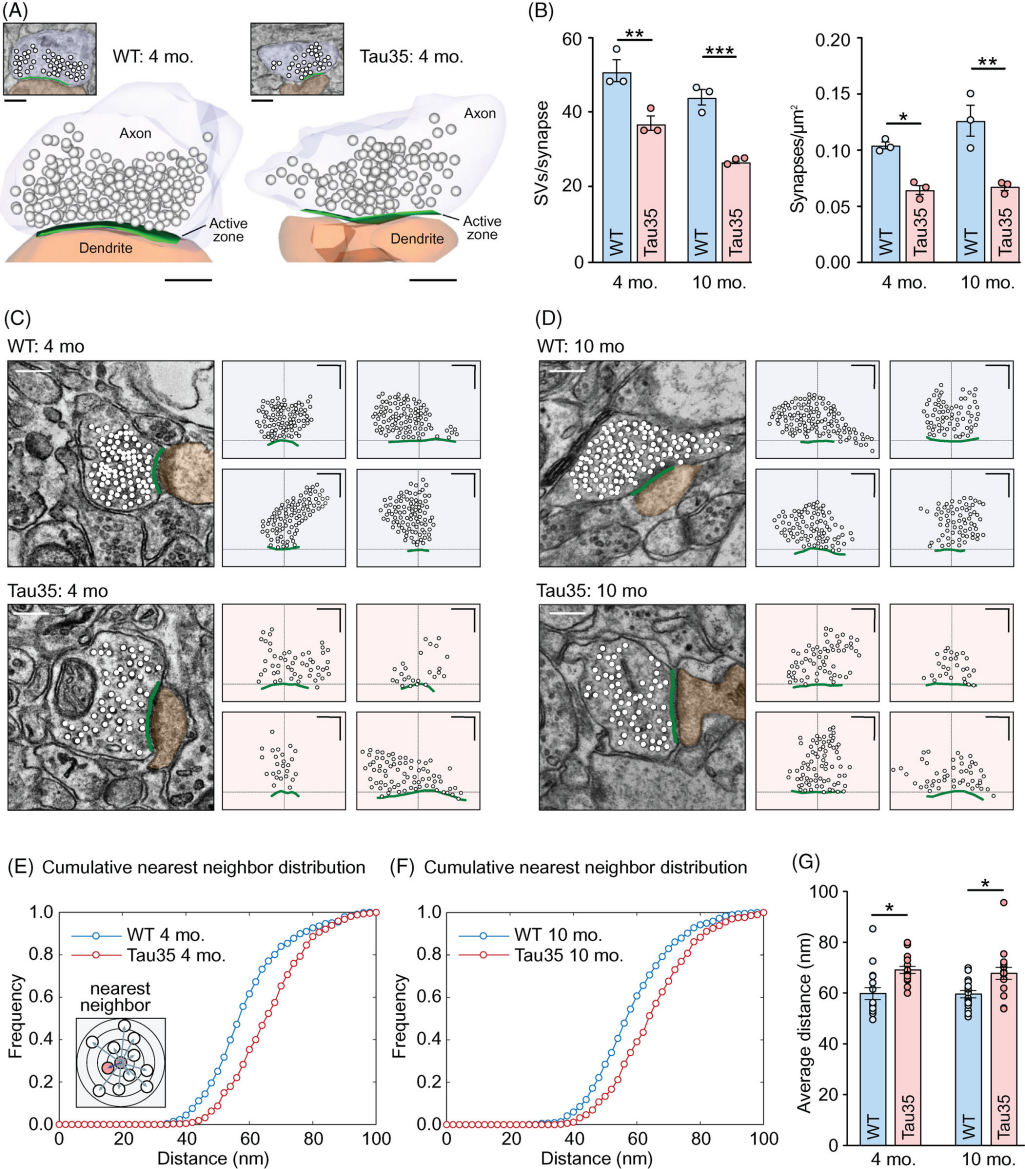
Ultrastructural analysis of synapses in WT and Tau35 mouse brain. A, Representative electron micrographs (top, inset) and 3D serial reconstructions of WT and Tau35 CA1 hippocampal synapses in mice aged 4 months, based on five middle sections through the synapse. Presynaptic (light blue) and postsynaptic (brown) structures are separated by the electron‐dense active zone (green). SVs are shown in white. Scale bars: 250 nm. B, Plots showing the number of SVs per synapse (left) and synaptic density (right) in the CA1 region of the hippocampus in WT and Tau35 mice aged 4 and 10 months. Two‐way ANOVA, *n* = 100 to 150 synapses from three mice of each genotype, ^*^ *P* < 0.05, ^**^ *P* < 0.01, ^***^ *P* < 0.001. C, D, Representative electron micrographs of CA1 synapses in WT and Tau35 mice aged 4 months (C) and 10 months (D) from randomly selected samples were used for analysis of spatial organization. Structures are pseudocolored: presynaptic terminal (light blue), postsynaptic terminal (brown), active zone (green), and SVs (white). Scale bars: 300 nm. Schematics of typical vesicle clusters in synaptic terminals in WT and Tau35 mice are shown on the right for each panel. Circles indicate individual SVs; green lines show active zones and crosshairs show active zone centers. Scale bars: 300 nm. E, F, Analysis of vesicle clustering based on nearest neighbor analysis. Main plots are cumulative frequency distributions of the distance to the nearest neighbor averaged across *n* = 16 synapses for each condition. Inset in (E) is a schematic illustrating the principle of measure. G, Plot shows mean ± SEM of the average distance between closest vesicle pairs for synapses from WT and Tau35 mice at 4 and 10 months old (*n* = 16 synapses in each group; total vesicle counts: 1210, 840, 1452, 801). Two‐way ANOVA, *P* < 0.01, asterisks indicate results of pairwise comparisons (Tukey multiple comparisons test, ^*^ *P* < 0.05). ANOVA, analysis of variance; CA, cornu ammonis; SVs, synaptic vesicles; SEM, standard error of the mean; WT, wild type.

We next used an ultrastructural approach to characterize the possible impact of Tau35 on presynaptic structure in the hippocampal CA1 region in mice (Figure 4A). In representative electron micrograph sections taken from mice aged 4 months, we recorded a significant reduction (27%) in the number of SVs per terminal in Tau35 versus WT hippocampus (Figure 4B). In Tau35 mice aged 10 months, this difference was even more pronounced (39% reduction in Tau35 mice, Figure 4B). In contrast, there were no significant changes in the presynaptic area, the diameter of SVs, or the percentage of docked SVs in Tau35 mice at either age (Figure S4A‐C in supporting information). These findings indicate that Tau35 has a marked and sustained negative impact on correlates of presynaptic strength that progress further with aging.

The reduction of synaptic vesicle numbers in Tau35 mice suggests possible alterations in the spatial organization of clusters between conditions. To examine this idea, we mapped vesicle positions in a randomly selected sample of electron micrographs that include the active zone (Figure 4C, D). A consistent feature of these maps was that vesicles appeared more loosely clustered in Tau35 mice at both age points. To quantify this observation, we carried out a proximity analysis by measuring the distance of each vesicle to its nearest neighbor and used these values to generate mean cumulative frequency distribution plots (Figure 4E, F). Compared to WT conditions, Tau35 synapses had a strikingly right‐shifted distribution at both time points supporting our observation that vesicles were less tightly associated. We confirmed this by comparing the average vesicle–vesicle distance for each synapse showing that the separation between nearest neighbors was ≈ 10 nm greater in Tau35 versus WT mice (Figure 4G). Taken together, our findings suggest that Tau35 influences key features of synaptic and vesicle organization, including the mechanisms that serve to cluster vesicles at terminals.

In addition to SV‐driven fast transmission, slower synaptic signaling occurs via neuropeptides packaged in dense‐core vesicles (DCVs). Therefore, we quantified the percentage of synapses harboring DCVs in Tau35 and WT mice. At 4 months of age, there was no difference between the genotypes (Figure S4D). However, 10‐month‐old Tau35 mice exhibited a marked (74%) reduction in the percentage of hippocampal synapses with DCVs (Figure S4D). This reduction in DCV‐containing synapses in Tau35 mice occurs at a time when overt cognitive dysfunction and tauopathy are apparent in these animals, suggesting that the lack of synaptic DCVs could result in compromised release of neuropeptides and thereby further inhibit information transfer in neural networks.

We next performed immunogold electron microscopy to determine whether HCN1 and HCN3 channels are present in the hippocampus of Tau35 and WT mice. We observed individual and clustered distributions of gold‐labeled HCN1 and HCN3 closely opposed to synapses in the CA1 region of both Tau35 and WT mouse hippocampus. These findings suggest a role for HCN1 and HCN3 channels in both presynaptic and postsynaptic functions (Figure S4E).

### 3.4 Tau35 influences *I*_h_‐dependent sag and excitatory postsynaptic current kinetics in hippocampal neurons

**FIGURE 5:**
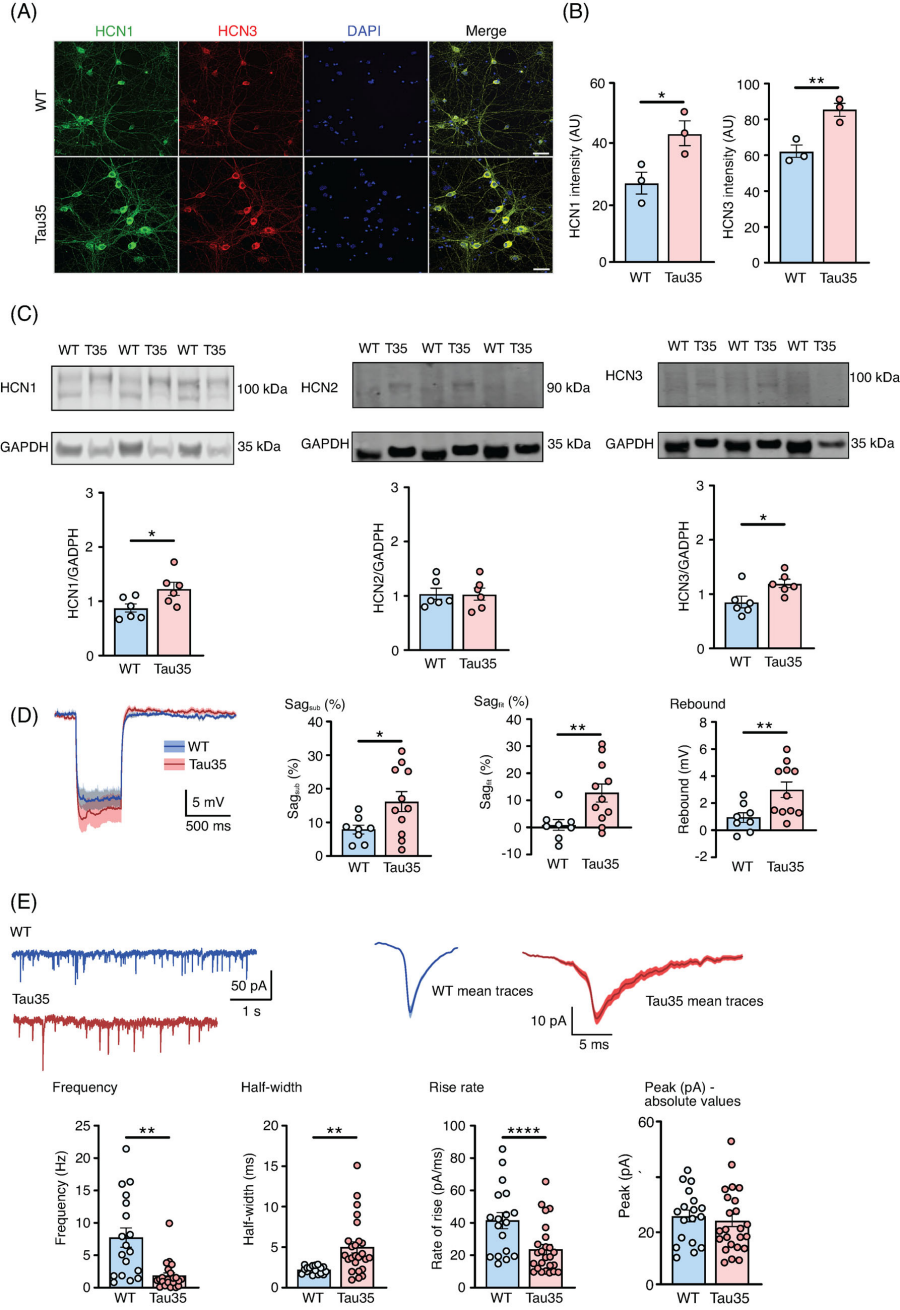
Increased HCN channel expression and functional changes in Tau35 hippocampal neurons. A, Immunofluorescence labeling of WT and Tau35 mouse hippocampal neurons at 14 DIV with HCN1 and HCN3 antibodies, and DAPI staining of nuclei. Scale bars: 10 µm. B, Graphs show quantification of mean fluorescence intensity (± SEM) of HCN1 and HCN3. *n* = 150 (WT) and *n* = 120 (Tau35) neurons, from three independent experiments. Student *t* test, ^*^ *P* < 0.05, ^**^ *P* < 0.01. C, Western blots of lysates of primary hippocampal neurons (14 DIV) from WT and Tau35 mice, probed with antibodies to HCN1 (left), HCN2 (middle), or HCN3 (right), and GAPDH. Quantification of the blots is shown in the graphs as mean ± SEM; *n* = 6 independent experiments. Student *t* test, ^*^ *P* < 0.05. D, Average traces ± SEM boundaries of *V*_m_ hyperpolarization by injection of ∐100 pA, 500 ms current in WT and Tau35 mouse hippocampal neurons (11 to 16 DIV). The recordings used a pre‐stimulus potential of −80 mV. Graphs show sag_sub_, sag_fit_, and rebound potential. Graphs show quantification of the mean ± SEM; *n* = 8 (WT) and *n* = 11 (Tau35) neurons. Student *t* test, *P < 0.05, ^**^ *P* < 0.01. E, sEPSCs in WT and Tau35 hippocampal neurons (11 to 16 DIV). Left panel: representative traces; right panel: average traces ± SEM. Graphs show the frequency, half‐width, rate of rise, and amplitude (absolute values) of sEPSCs. Graphs show quantification of the mean ± SEM; *n* = 18 (WT) and *n* = 25 (Tau35) neurons. Student *t* test, ^**^ *P* < 0.01, ^****^ *P* < 0.0001. DAPI, 4′,6‐diamidino‐2‐phenylindole; DIV, days in vitro; GAPDH, glyceraldehyde 3‐phosphate dehydrogenase; HCN, hyperpolarization‐activated cyclic nucleotide‐gated; SEM, standard error of the mean; sEPSCs, Spontaneous excitatory postsynaptic currents; WT, wild type.

To look for functional correlates of morphological deficits in Tau35 neurons, we performed a detailed electrophysiological analysis using hippocampal neuronal cultures. First, to confirm the validity of this in vitro preparation, we used immunofluorescence labeling to show that all four HCN channels are expressed at 14 DIV (Figure 5A and Figure S5A in supporting information). Strikingly, the same general pattern of increased HCN channel expression was seen in these cultured neurons as we observed in the brains of human AD and Tau35 mice (Figures 1 and 2). Specifically, there were significant increases in both HCN1 (59%) and HCN3 (38%) in Tau35 neurons versus WT (Figure 5B), but no differences in HCN2 or HCN4 channel expression (Figure S5A). Likewise, quantification of HCN1 and HCN3 channels on western blots of Tau35 hippocampal neurons showed increases of 41% and 40% respectively, with no change in HCN2 channel expression (Figure 5C). Coupled with the increase in tau phosphorylation (Figure S3E), our in vitro model therefore recapitulates the key features of human AD and Tau35 mice and is thus a disease‐relevant tool in which to assess functional changes.

Next, to establish the effects of Tau35 on neuronal excitability in early development, we performed whole‐cell recordings from cultured hippocampal neurons at 11 to 16 DIV. Specifically, we recorded the deflection of the plasma membrane voltage (*V*_m_) in response to a hyperpolarizing current step to explore the *I*_h_‐dependent sag and rebound^23^ properties in Tau35 and WT neurons (Figure 5D). Sag was quantified by the subtraction of the negative peak (sag_sub_) and of the extrapolated first exponential fit of the *V*_m_ decay (sag_fit_) upon the injection of a hyperpolarizing step^23^ (illustrated in Figure S5B). Sag_sub_ increased approximately 2‐fold and sag_fit_ was elevated almost 15‐fold in Tau35 versus WT hippocampal neurons. The increased sag was accompanied by a 3‐fold rise in rebound potential in Tau35 neurons when the hyperpolarizing current step ended (Figure 5D). Because sag potentials increase in response to activation of *I*_h_ current through HCN channels,^29^ our data strongly suggest that the augmented sag in Tau35 neurons is due to increased expression of HCN1 and HCN3 channels (Figure 5A‐C). Notably, in primary cortical neurons, despite the increased expression of phosphorylated tau and HCN3 channels (Figure S5C‐D) we detected no changes in sag voltage (Figure S5E), suggesting a cell type–specific role of HCN channels. In addition, in primary hippocampal neurons, no changes were observed in the biophysical properties of the currents generated by non‐inactivating potassium channels, including the maximal conductance (*G*_max_) and the half‐activation voltage (*V*_1/2_) in Tau35 hippocampal neurons (Figure S5F‐G), highlighting HCN specific effects on these neurons.

To investigate whether the changes that we attribute to HCN channels affected downstream information signaling, we next recorded sEPSCs in Tau35 and WT neurons at 11 to 16 DIV (Figure 5E). We found that Tau35 neurons exhibited a marked reduction (77%) in their sEPSC frequency (Figure 5E), without changes in amplitude (Figure 5E and Figure S5H), consistent with Tau35 reducing the probability of presynaptic vesicle release. The half‐width of sEPSCs in Tau35 neurons increased more than 2‐fold, and the rate of rise reduced to 43% compared to WT neurons (Figure 5E), which may contribute to temporally less precise spike responses.^30^ Given the established link between asynchronous vesicle release and the timing of sEPSCs^31^, ^32^ we propose that these Tau35‐associated changes in sEPSC kinetics might reflect changes in SV recycling.

Finally, to assess the effect of Tau35 on the electrogenic properties of the plasma membrane, we evoked action potentials using a 300 pA, 500 ms square current injection and measured their waveform properties (Figure S5F). None of the action potential properties measured, including peak, threshold, maximum rate of rise, peak width, sEPSC amplitude, time constant (tau), or rate of decay were affected by Tau35 (Figure 5E, Figure S5G‐H and data not shown). Our findings of decreased frequency and increased sEPSC width, while the amplitude remains unaffected, therefore suggest that decreased synaptic and vesicular density lead to functional alterations, but compensatory postsynaptic adjustments may intervene, resulting in no change in sEPSC amplitude.

## 4 DISCUSSION

**FIGURE 6:**
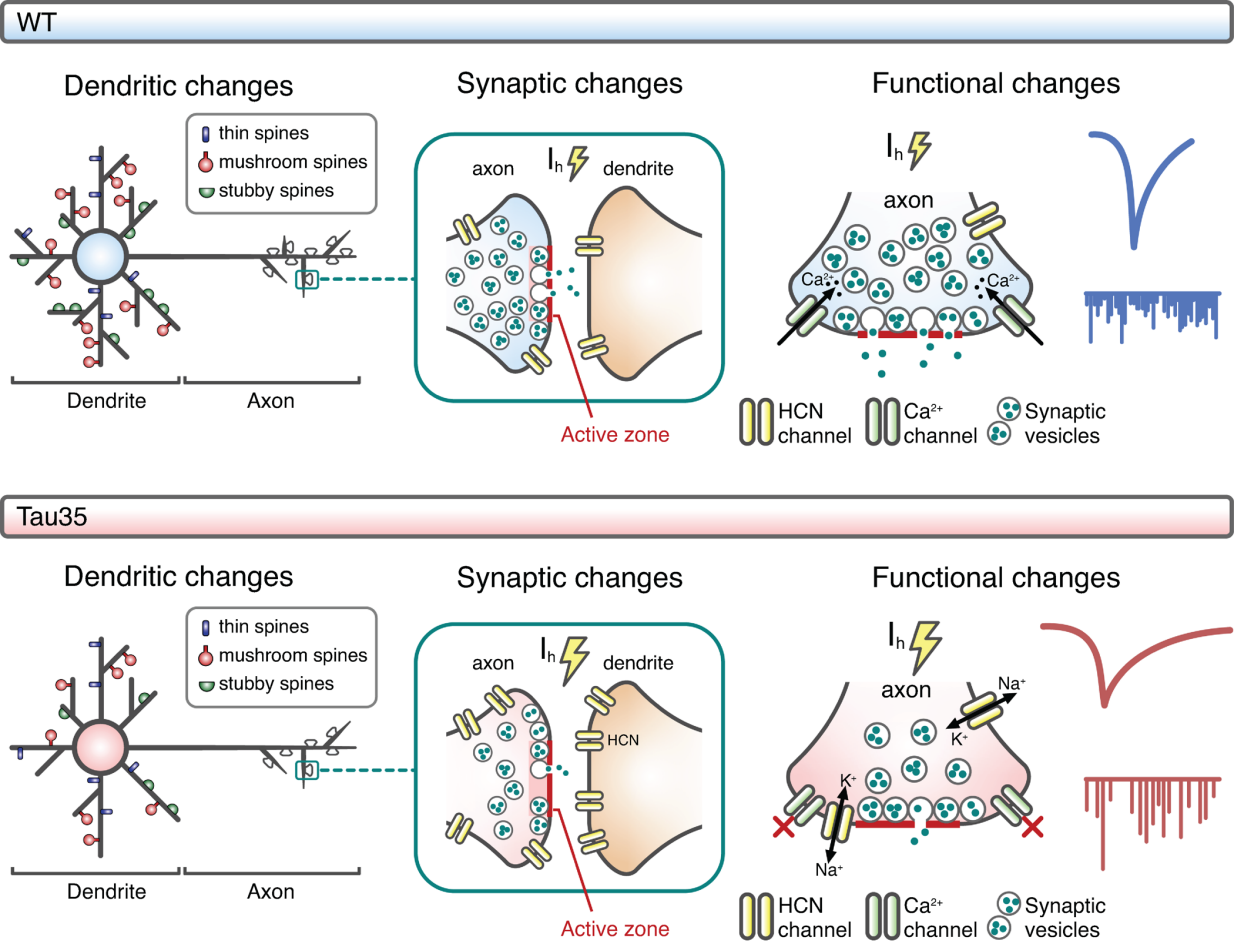
Proposed model of Tau35‐induced hyperpolarization‐activated cyclic nucleotide‐gated (HCN) channelopathy and synaptic dysfunction in the hippocampal area. Expression of Tau35 results in reduced dendritic complexity and spine density, accompanied by alterations at synapses, including decreased synaptic vesicle density, and looser clustering. Increased expression of HCN channels is paralleled by increases in *I*_h_‐dependent sag voltage and half‐width of spontaneous excitatory postsynaptic currents (sEPSCs), along with reductions in the frequency and rate of rise of sEPSCs. *I*_h_, hyperpolarization‐activated inward current; WT, wild‐type.

Proper maintenance of synaptic structure and function is critical for cognitive processing, and synaptic dysfunction is an early correlate of dementia and related tauopathies.^33^ However, the molecular mechanisms that underlie the synaptic damage caused by tau deposition are not understood. Here we demonstrate pathological alterations in the expression of selected HCN channels in human AD and Tau35 mouse brain that provide a basis for explaining wide‐ranging deficits in neuronal signaling and connectivity (Figure 6).

HCN channels regulate a range of cellular properties, including membrane resistance, intrinsic membrane excitability, and synaptic integration,^6^ and are emerging as key players in the pathogenesis of several neurodegenerative diseases, including AD, Parkinson's disease, and amyotrophic lateral sclerosis.^13^ Interestingly, presynaptic and postsynaptic expression and localization of HCN1 channels are differentially regulated, with presynaptic HCN1 channel localization and function being independent of TRIP8b.^34^ Therefore, HCN channel localization could differentially impact neural network activity and excitability. Indeed, presynaptic HCN1 expression in perforant path axons is regulated by network activity and contributes to hippocampal maturation.^35^ Moreover, HCN1 channels in the active zone of mature glutamatergic cortical synaptic terminals regulate neurotransmission in the entorhinal cortex and thus contribute to memory formation and spatial navigation.^12^ Expression of HCN channels also determines *I*_h_ current generation and fine tunes communication between hippocampal subregions and cortical or subcortical networks.^36^, ^37^ As HCN channels regulate hippocampus‐dependent learning and memory,^38^ the imbalance of HCN channel expression in AD and Tau35 mouse brain is a likely candidate for driving abnormal communication between hippocampal and cortical structures that triggers changes in memory formation and retrieval.

Changes in HCN1 channels, such as increases in *I*_h_ current and sag voltage, have been reported in several familial frontotemporal dementia and/or AD transgenic animal models, all of which overexpress mutant forms of human genes *APP, MAPT*, and/or *PSEN1*.^14^, ^15^, ^16^ However, to investigate human tauopathies that are not caused by *MAPT* overexpression, and to avoid experimental artifacts, it is important to characterize HCN channel–associated synaptic changes in a transgenic mouse model in which total tau is expressed at an endogenous level, such as Tau35 mice.^17^, ^39^ Progressive increases in HCN1 and HCN3 ion channel expression are accompanied by an array of dendritic, synaptic, and ultrastructural changes as well as the development of tau pathology in Tau35 mice, with decreases in dendritic branching observed only during advanced tauopathy. We note that expression of P301L mutant tau in rTg4510 transgenic mice results in a similar, robust reduction in dendritic arborization at the late stages of the disease.^15^ In the same P301L tau transgenic mouse, structural and functional synaptic alterations were observed in the early stages of tauopathy both in vivo^40^ and in vitro.^41^ Our findings have important mechanistic implications for disease because the degree of dendritic branching influences the generation of new synapses, neuronal function, and cognitive performance, all of which are impaired in tauopathies. Dendrites are also a major source of neuropeptides and their release, even independently of electrical activity, enables sustained re‐organization of neuronal networks.^42^ Our observation of a marked reduction in synapses harboring DCVs in the hippocampus of aged Tau35 mice also mirrors the reduction in hippocampal DCV peptide density reported in AD.^43^

Recent studies have shown that pathogenic tau alterations in axons precede disease‐associated changes in the somatodendritic compartment.^44^ Indeed, in the hippocampus of young presymptomatic Tau35 mice, we found enhanced phosphorylated tau load as well as marked reductions in synapse density and in the number of SVs in synaptic terminals, changes that precede the appearance of overt tau pathology in these animals.^17^ We also observed altered presynaptic cytoarchitecture in the brains of young Tau35 mice, with evidence for a looser distribution of SVs in terminals. We hypothesize that these changes could be associated with alterations in vesicle tethering, reducing their normally tight linkage in clusters near the active zone and allowing individual vesicles to leave the bouton and enter flanking axonal regions, thus reducing overall presynaptic vesicle counts. Synapsin, a key SV linker,^45^, ^46^ which we show has reduced expression in Tau35 neurons, could potentially contribute to this observed phenotype: synapsin knockout mice show analogous reductions in vesicle numbers in hippocampal terminals and increased clustering of vesicles in extrasynaptic regions.^21^, ^47^ Nonetheless, we note that the human AD samples did not show the same significant decrease in synapsin expression, suggesting that the influence of this protein is only likely to be a partial explanation for the observed phenotype. Other candidate substrates could include the SV‐associated protein tomosyn‐1, which is known to have roles in vesicle pool partitioning and clamping,^48^ as well as actin and other dynamic structural elements, which play important roles in trafficking vesicles between terminals.^49^, ^50^ Vesicle organization at presynaptic terminals of hippocampal synapses has been linked to synaptic efficacy,^51^, ^52^, ^53^ suggesting that disruptions in the integrity of SV clusters could underlie deficits in synaptic signaling in Tau35 mice.

Changes in the expression of voltage‐gated channels encoded by the HCN1–4 gene family have been reported in multiple neurological disorders.^13^ These channels have major roles in controlling synaptic transmission, integration of synaptic input, and neuronal excitability.^54^ In particular, HCN1 has been shown to mis‐localize and become dysfunctional in models of dementia.^14^, ^41^ Impaired trafficking of HCN1 channels to distal dendrites or increased HCN expression^14^, ^55^ is associated with increased *I*_h_‐dependent sag potential in multiple transgenic models of AD^14^, ^15^, ^16^ and increased sag amplitude has been detected in human layer 5 pyramidal neurons from older adults,^56^ suggesting a possible role for HCN in the development of disease.

We used cultured Tau35 hippocampal neurons to better understand how changes in the expression of HCN channels may drive dynamic structural and functional synaptic changes. Dendritic spine morphology in cultured Tau35 hippocampal neurons is important because activity‐dependent spine remodeling is critical for neuronal communication^57^ and synaptic strength is regulated by the number and size of individual dendritic spines.^26^, ^27^ In Tau35 hippocampal neurons we observed reduced density of dendritic spines and loss of mature spines, which adversely affects synaptic strength and further supports the view that tau truncation compromises synaptic communication.

Reduced dendritic and synaptic complexity have been linked to altered resonance in hippocampal pyramidal neurons of rTg4510 mice overexpressing P301L tau.^15^, ^41^ Similarly, we reported changes in resonance in Tau35 mice, including reduced impedance in older animals upon injection of an oscillating current.^39^ Here we observed altered membrane dynamics in Tau35 primary hippocampal neurons and identified increased voltage sensitivity of non‐inactivating potassium channels as a possible cause of the decreased impedance and voltage‐dependent input resistance caused by Tau35 expression. Conversely, Tau35 hippocampal neurons did not exhibit changes in either input resistance or biophysical properties of voltage‐gated potassium channels that generate M‐currents (*I*_M_).^58^ However, we did detect increased *I*_h_‐dependent sag of the *V*_m_ deflection upon injection of negative current, paralleled by elevations in the *I*_h_‐generating HCN1 and HCN3 channels. Interestingly, both *I*_h_ and *I*_M_ currents play critical roles in regulating plasma membrane excitability in the presence of oscillating potentials, driving neuronal resonance.^59^ The interplay of *I*_h_ and *I*_M_ has recently been reported to regulate intrinsic neuronal excitability and to affect disease progression in a mouse model of amyotrophic lateral sclerosis.^60^ Neurons with strong *I*_h_ currents are good resonators and operate as bandwidth filters.^61^, ^62^ In contrast, inhibited *I*_h_ and increased *I*_M_ current results in low resonance, causing the neuron to function as a low‐pass filter and attenuating higher frequencies.^63^ Our data demonstrate that Tau35 is associated with increased *I*_h_‐related events in primary hippocampal neurons. We previously performed electrophysiological recordings in brain slices from older Tau35 mice and found increased *I*_M_ current.^39^ Notably, any alteration to the *I*_h_:*I*_M_ ratio caused by Tau35 expression could interfere with intrinsic neuronal excitability and the ability of hippocampal neurons to respond to specific stimuli, further disrupting neuronal connectivity.

Tau35 primary hippocampal neurons developed sEPSCs that were less frequent, wider, and with slower rise times than those of WT neurons. These currents reflect alterations in presynaptic glutamate release and subsequent activation of postsynaptic AMPA and NMDA receptors.^64^ Notably, fluctuation in the release of presynaptic vesicles has been reported to profoundly affect both the timing of sEPSCs^31^, ^32^ and SV distribution along the active zone.^65^ Therefore, the changes we observed in sEPSCs in Tau35 hippocampal neurons may underline a causal relationship between reduced synaptic activity and asynchronous vesicle recycling.

Finally, key action potential properties, such as peak, threshold, maximum rate of rise, peak width, EPSC amplitude, time constant (tau), and rate of decay, as expected, were not affected by Tau35 expression. We are not aware of any direct relationship between HCN expression and action potential properties, except in very specific instances. Such an example is pacemaker cells, in which HCN channels are the major drivers of the repolarization leading to a new action potential after hyperpolarization. In the hippocampal CA1 network, where this study is focused, HCN channels are primarily involved in driving the sub‐threshold resonant properties of the cell. As such, HCN channels are involved in H‐resonance, which enables neurons to act as a bandwidth filter at hyperpolarized potentials.^66^

In conclusion, this correlative study suggests previously unreported roles for HCN channels in the development and progression of tauopathy. Our results demonstrate that increased expression of HCN channels in the hippocampus is likely responsible for the increased sag voltage apparent in Tau35 neurons. The aberrant signaling induced by Tau35 coincides with changes in synaptic ultrastructure, reducing the density of synaptic terminals as well as the clustering of SVs in individual terminals, adversely affecting network connectivity. Our results suggest that selective targeting of specific HCN channels with pharmacological agents could potentially protect synapses and prevent disease progression in human tauopathy.

## CONFLICT OF INTEREST STATEMENT

The authors declare no competing interests. Author disclosures are available in the supporting information.

## CONSENT STATEMENT

All studies were approved by the institutional review boards of King's College London and the MRC London Neurodegenerative Diseases Brain Bank. *Post mortem* human tissue used in this study was obtained on the basis of informed consent.

## Supporting information

Supporting Information

Supporting Information

Supporting Information

Supporting Information

Supporting Information

Supporting Information
