# Supplementary material for: Tau‐mediated synaptic dysfunction is coupled with HCN channelopathy
Source: Alzheimers Dement. 2024 Jul 12;20(8):5629–46. doi: 10.1002/alz.14074 (PMC11350046; doi:10.1002/alz.14074)
Supplement: Supplementary file 1 — Supporting Information [file ALZ-20-5629-s002.pdf]

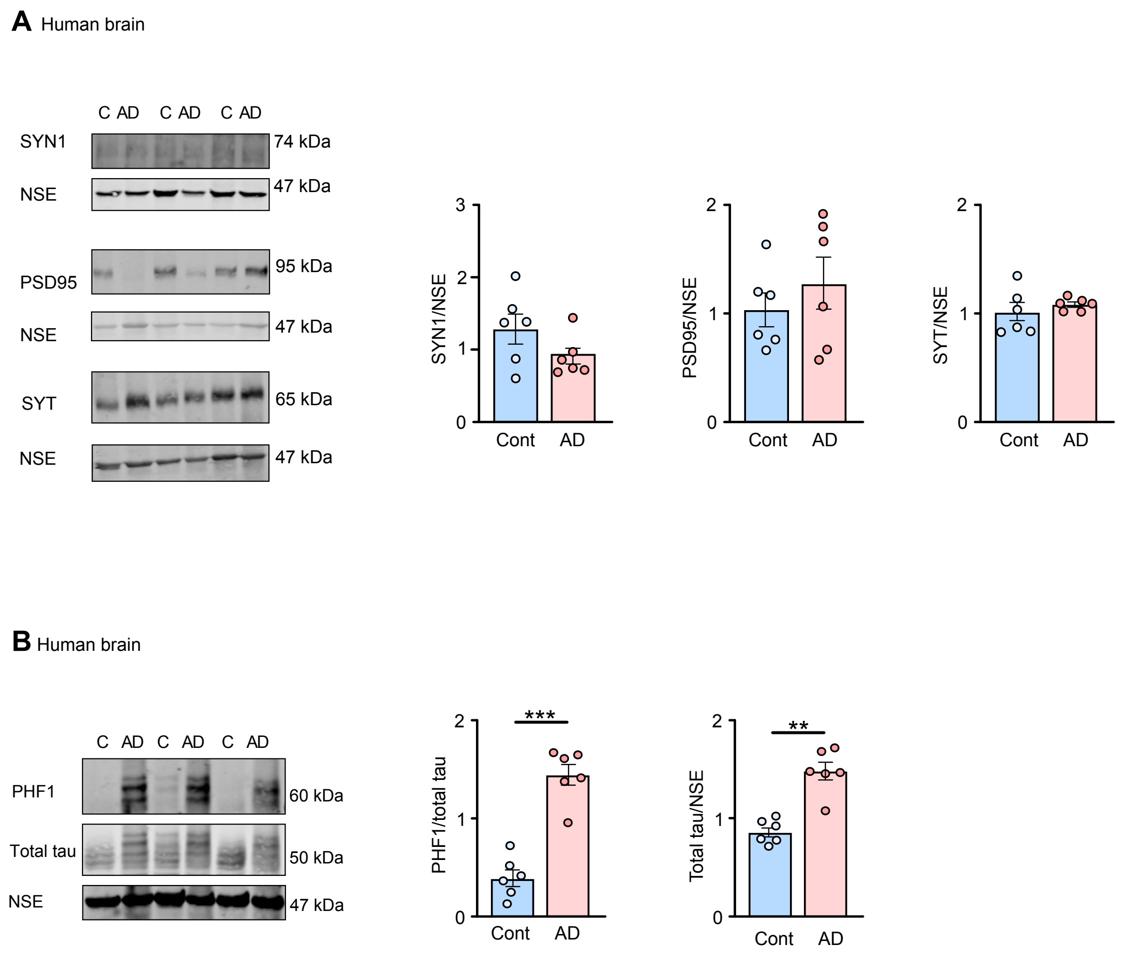

Supplementary Figure 1. Analysis of phosphorylated tau and synaptic markers in human *post-mortem* AD hippocampus

A, Western blots of hippocampal homogenates from AD and control *post-mortem* human brain probed with antibodies to SYN1, PSD95, SYT, and NSE. **B**, Phosphorylated tau (PHF-1), total tau, and NSE. The graphs show quantification of total tau normalized to NSE and the ratio of phosphorylated tau to total tau, mean ± SEM; n= 6 brains per group. Student *t* test, $^{**}P < 0.01$. AD, Alzheimer's disease; SYN1, synapsin 1; PSD95, postsynaptic density 95; SYT, synaptotagmin; NSE, neuron specific enolase; SEM, standard error of the mean
