# Supplementary material for: Tau‐mediated synaptic dysfunction is coupled with HCN channelopathy
Source: Alzheimers Dement. 2024 Jul 12;20(8):5629–46. doi: 10.1002/alz.14074 (PMC11350046; doi:10.1002/alz.14074)
Supplement: Supplementary file 2 — Supporting Information [file ALZ-20-5629-s005.pdf]

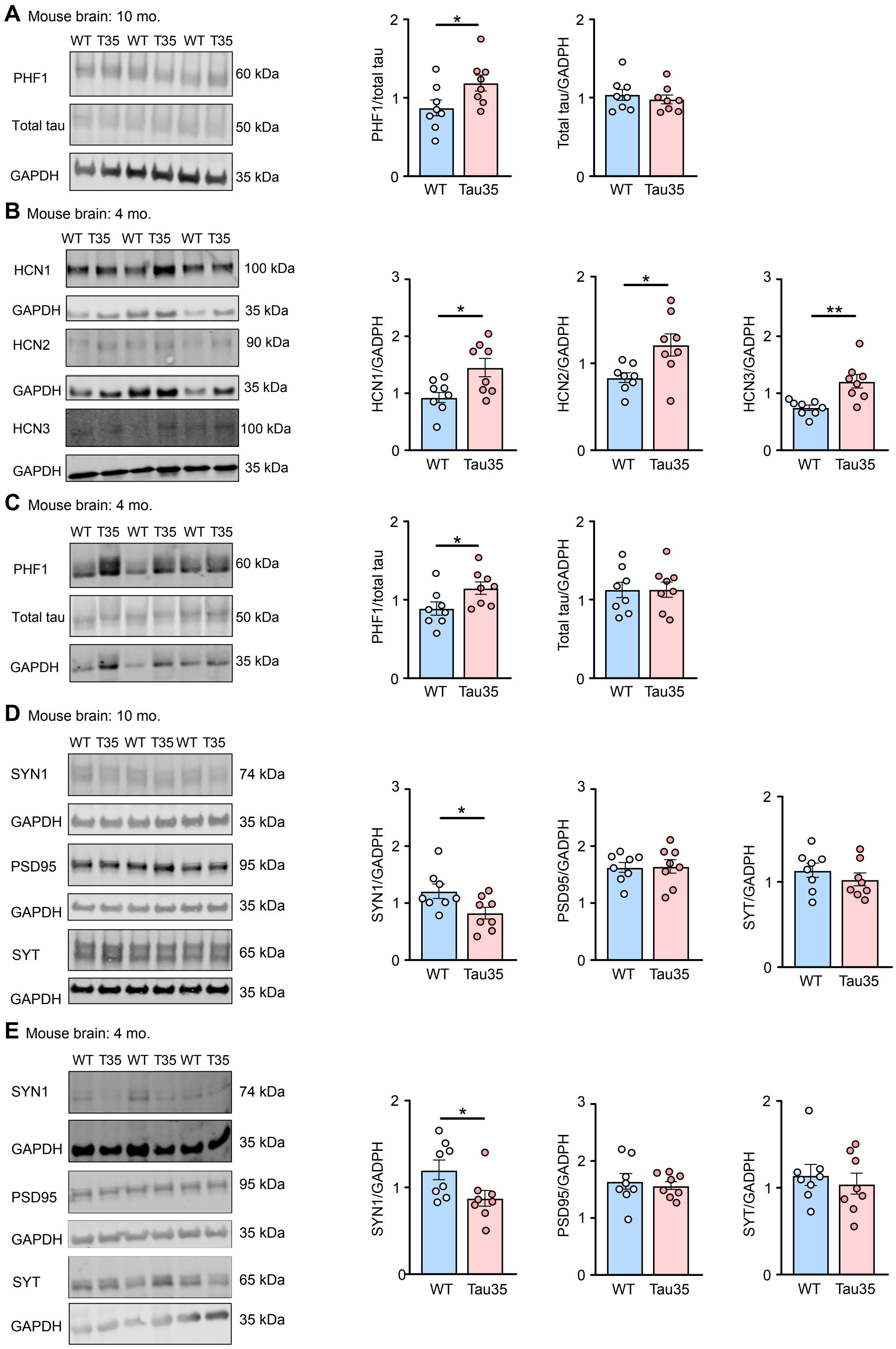
A Mouse brain: 10 mo.
WT T35 WT T35 WT T35
PHF1
60 kDa
Total tau
50 kDa
GAPDH
35 kDa
PHF1/total tau
Total tau/GADPH
WT Tau35
B Mouse brain: 4 mo.
HCN1
100 kDa
HCN2
90 kDa
HCN3
HCN1/GADPH
HCN2/GADPH
HCN3/GADPH
C Mouse brain: 4 mo.
D Mouse brain: 10 mo.
SYN1
74 kDa
PSD95
95 kDa
SYT
65 kDa
SYN1/GADPH
PSD95/GADPH
SYT/GADPH
E Mouse brain: 4 mo.

Supplementary Figure 2. Analysis of synaptic markers in WT and Tau35 brain homogenates

A, Western blots of hippocampal homogenates from WT and Tau35 mice aged 10 months, probed with antibodies to phosphorylated tau (PHF-1), total tau and GAPDH. **B,** Western blots of hippocampal homogenates from WT and Tau35 mice aged 4 months (pre-symptomatic) probed with antibodies to HCN1, HCN2, HCN3) and GAPDH. **C,** Western blots of hippocampal homogenates from WT and Tau35 mice aged 4 months (pre-symptomatic) probed with antibodies to phosphorylated tau (PHF-1), total tau and GAPDH. **D,E**, Western blots of hippocampal homogenates from WT and Tau35 mice, aged 10 months and 4 months respectively, probed with antibodies to SYN1, SYT, PSD95 and GAPDH. Quantification of the blots is shown in the graphs as mean ± SEM; n= 8 brains per group. Student *t* test, **P* < 0.05, ***P* < 0.01. WT, wild-type; GAPDH, glyceraldehyde 3-phosphate dehydrogenase; HCN, hyperpolarization-activated cyclic nucleotide-gated; SYN1, synapsin 1; SYT, synaptotagmin; PSD95, postsynaptic density 95; SEM, standard error of the mean.
