# Supplementary material for: Tau‐mediated synaptic dysfunction is coupled with HCN channelopathy
Source: Alzheimers Dement. 2024 Jul 12;20(8):5629–46. doi: 10.1002/alz.14074 (PMC11350046; doi:10.1002/alz.14074)
Supplement: Supplementary file 3 — Supporting Information [file ALZ-20-5629-s003.pdf]

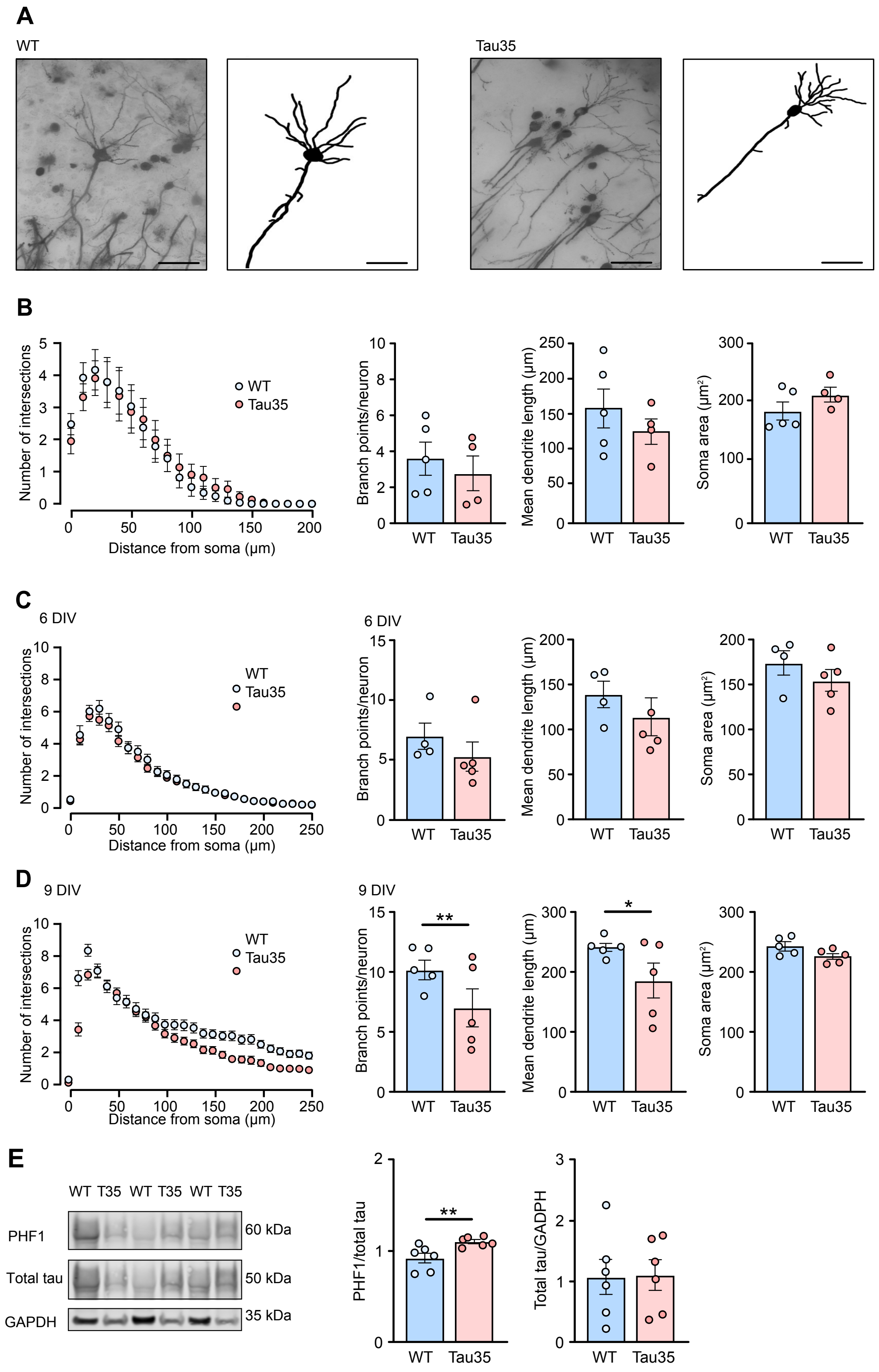
A
WT
Tau35
B
Number of intersections
WT
Tau35
Distance from soma (µm)
Branch points/neuron
Mean dendrite length (µm)
Soma area (µm²)
WT Tau35
C
6 DIV
Number of intersections
WT
Tau35
Distance from soma (µm)
6 DIV
Branch points/neuron
Mean dendrite length (µm)
Soma area (µm²)
D
9 DIV
Number of intersections
WT
Tau35
Distance from soma (µm)
9 DIV
Branch points/neuron
Mean dendrite length (µm)
Soma area (µm²)
E
WT T35 WT T35 WT T35
PHF1
Total tau
GAPDH
60 kDa
50 kDa
35 kDa
PHF1/total tau
Total tau/GADPH
WT Tau35

Supplementary Figure 3. Dendritic branching in young Tau35 mice and Tau35 hippocampal neurons

A, Images of Golgi-Cox stained WT and Tau35 CA1 hippocampal neurons in the brains of 4-month-old mice. Scale bars: 50µm. **B**, Total Sholl analysis shows no differences in basal dendrite complexity in neurons of WT and Tau35 mice aged 4 months. Graph shows quantification of the mean ± SEM, n=40 neurons from 4 to 5 mice of each genotype. Two-way ANOVA, $P > 0.05$. Graphs show the number of primary branch points, dendrite length and soma area in neurons of WT and Tau35 mice aged 4 months, n=40 neurons from 4 to 5 mice of each genotype. Student t test, $P > 0.05$. **C**, Sholl analysis of dendritic branching in WT and Tau35 primary hippocampal neurons at 6 days *in* vitro. Graph shows mean ± SEM. Two-way ANOVA ($P < 0.01$), n=50 neurons of each genotype from 5 independent experiments. Graphs below show mean ± SEM number of dendritic branch points, mean dendrite length and soma area, n=39-52 neurons from 4-5 independent experiments. Student t test, $P > 0.05$. **D**, Sholl analysis of dendritic branching in WT and Tau35 hippocampal neurons at 9 DIV. Graph shows mean ± SEM. Two-way ANOVA ($P < 0.01$), n=55 neurons of each genotype from 5 independent experiments. Graphs below show mean ± SEM of the number of dendritic branch points, mean dendrite length and soma area, n=55 neurons of each genotype from 5 independent experiments. Student t test, *$P < 0.05$, **$P < 0.01$. **E**, Western blots of lysates of primary hippocampal neurons (14 DIV) from WT and Tau35 mice, probed with antibodies to phosphorylated tau (PHF-1), total tau and GAPDH. Quantification of the blots is shown in the graphs as mean ± SEM; n=4 independent experiments. Student's t-test, **$P < 0.01$. WT, wild-type; GAPDH, glyceraldehyde 3-phosphate dehydrogenase; SEM, standard error of the mean.
