# Supplementary material for: Tau‐mediated synaptic dysfunction is coupled with HCN channelopathy
Source: Alzheimers Dement. 2024 Jul 12;20(8):5629–46. doi: 10.1002/alz.14074 (PMC11350046; doi:10.1002/alz.14074)
Supplement: Supplementary file 4 — Supporting Information [file ALZ-20-5629-s001.pdf]

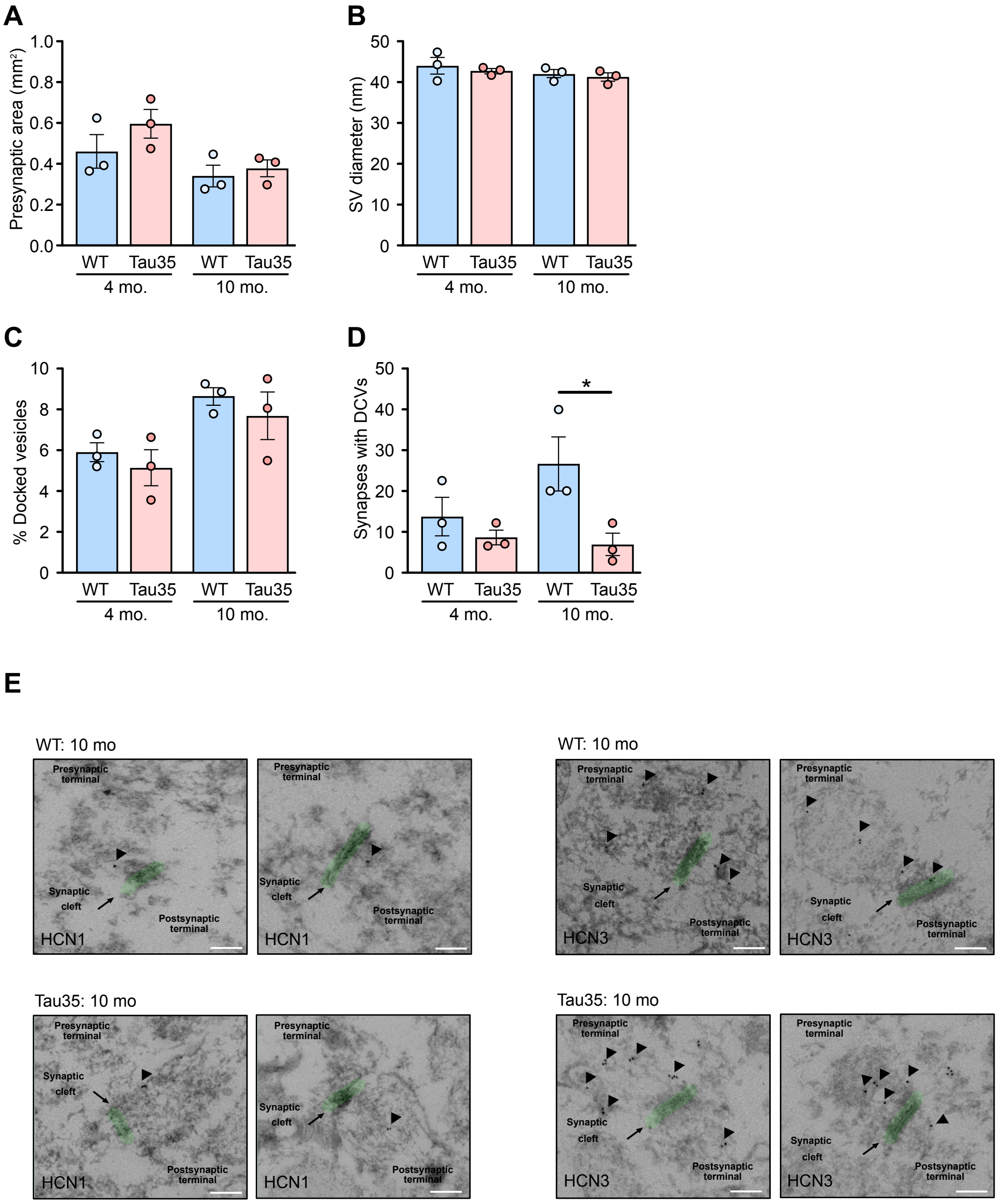

A
Presynaptic area (mm²)
1.0
0.8
0.6
0.4
0.2
0.0
WT
Tau35
WT
Tau35
4 mo.
10 mo.
B
SV diameter (nm)
50
40
30
20
10
0
WT
Tau35
WT
Tau35
4 mo.
10 mo.
C
% Docked vesicles
10
8
6
4
2
0
WT
Tau35
WT
Tau35
4 mo.
10 mo.
D
Synapses with DCVs
50
40
30
20
10
0
*
WT
Tau35
WT
Tau35
4 mo.
10 mo.
E
WT: 10 mo
Presynaptic terminal
Synaptic cleft
Postsynaptic terminal
HCN1
HCN3
Tau35: 10 mo

Supplementary Figure 4. Ultrastructural analysis of wild-type and Tau35 mouse hippocampal synapses

A,B, Quantification of SV diameter and presynaptic area of synapses in the CA1 region of the hippocampus in WT and Tau35 mice aged 4 and 10 months. **C**, Graphs show the percentage of docked vesicles per synapse in WT and Tau35 mice aged 4 and 10 months. **D**, Graphs show the percentage of synapses harbouring dense core vesicles in WT and Tau35 mice aged 4 and 10 months. For **A to D**: Graphs show mean ± SEM, *n*=100-150 synapses from 3 mice of each genotype. Two-way ANOVA, *$P < 0.05$. **E**, Immunogold electron microscopy of CA1 hippocampal Tau35 and WT control brain tissue (mice aged 10 months). Immunogold labeling by HCN1 and HCN3 antibodies shows synaptic localization of HCN1 (left panels) and HCN3 (right panels) channels (arrowheads). In each panel, the green line in the middle designates the synaptic cleft. Both HCN1 and HCN3 show individual and clustered distributions closely opposed to synapses in the CA1 region of Tau35 and WT mouse hippocampus. Scale bars: 200 nm. SV, synaptic vesicle; WT, wild-type; CA, cornu ammonis; HCN, hyperpolarization-activated cyclic nucleotide-gated; SEM, standard error of the mean.
