# Supplementary material for: Tau‐mediated synaptic dysfunction is coupled with HCN channelopathy
Source: Alzheimers Dement. 2024 Jul 12;20(8):5629–46. doi: 10.1002/alz.14074 (PMC11350046; doi:10.1002/alz.14074)
Supplement: Supplementary file 5 — Supporting Information [file ALZ-20-5629-s004.pdf]

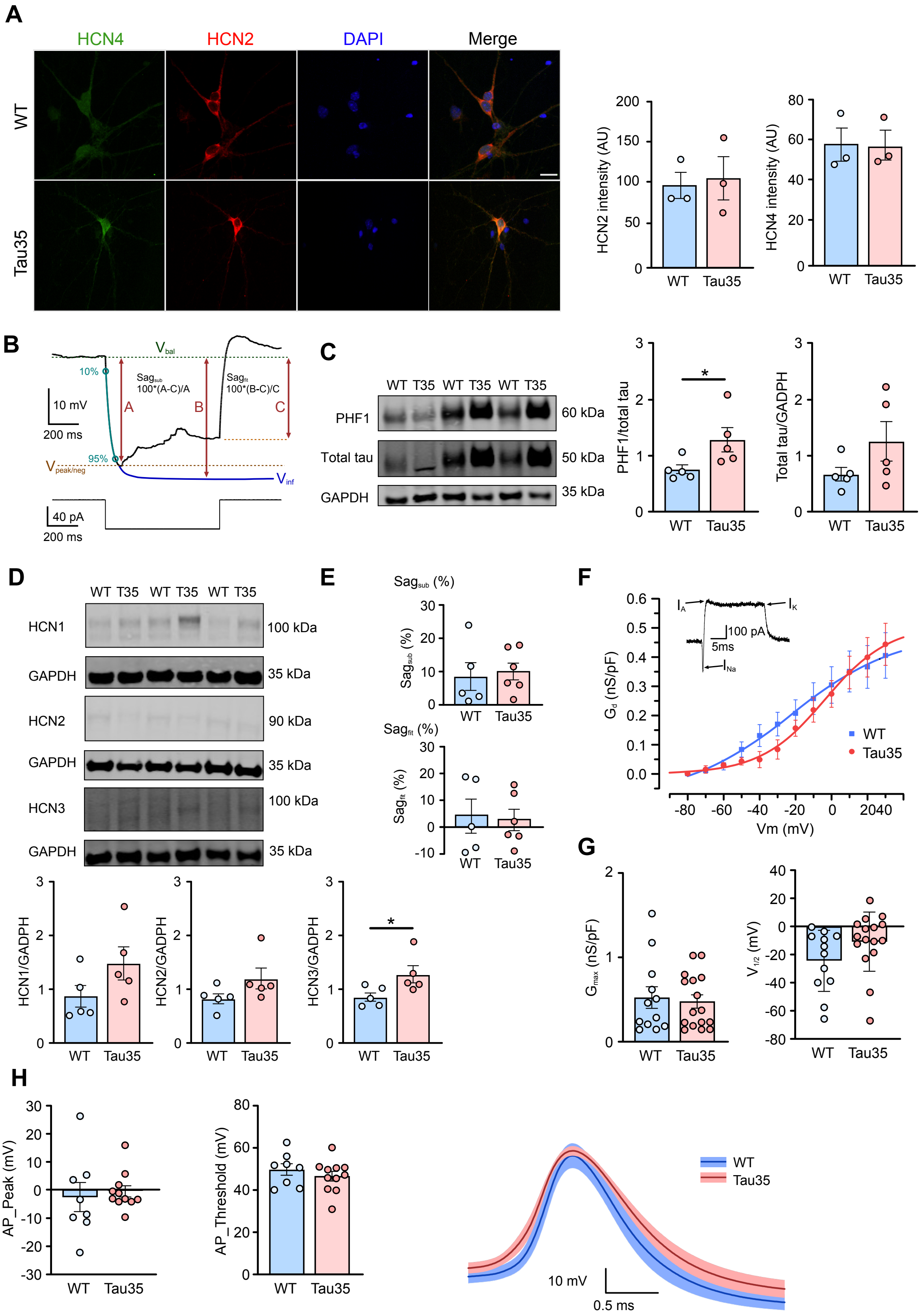
A
HCN4
HCN2
DAPI
Merge
WT
Tau35
HCN2 intensity (AU)
HCN4 intensity (AU)
WT
Tau35
B
Vbal
10%
95%
Sagsub
100*(A-C)/A
Sagfit
100*(B-C)/C
A
B
C
10 mV
200 ms
Vpeak/neg
Vinf
40 pA
200 ms
C
WT T35 WT T35 WT T35
PHF1
Total tau
GAPDH
60 kDa
50 kDa
35 kDa
PHF1/total tau
Total tau/GADPH
D
HCN1
HCN2
HCN3
GAPDH
100 kDa
35 kDa
90 kDa
HCN1/GADPH
HCN2/GADPH
HCN3/GADPH
E
Sagsub (%)
Sagfit (%)
F
IA
IK
INa
100 pA
5ms
Gd (nS/pF)
Vm (mV)
WT
Tau35
G
Gmax (nS/pF)
V1/2 (mV)
H
AP_Peak (mV)
AP_Threshold (mV)
10 mV
0.5 ms

Supplementary Fig 5. I_k conductance and action potential characteristics in Tau35 hippocampal neurons

A, Immunofluorescence labelling of WT and Tau35 mouse hippocampal neurons at 14 days *in* vitro (DIV) with HC2 and HCN4 antibodies. Scale bars: 10µm. Graphs show quantification of mean fluorescence intensity (± SEM) of HCN2 and HCN4. n=150 (WT) and n=120 (Tau35) neurons, from 3 independent experiments, Student *t* test, *P*>0.05. **B**, Schematic representation of the deflection of the plasma membrane voltage in response to a hyperpolarizing current step (500 ms, -100 pA) to explore I_h-dependent sag properties. The steady state voltage deflection (A) and the voltage extrapolated *ad infinitum* (B) for a single exponential function fit between 10% and 90% of the minimum point (C) of the voltage deflection, were used to calculate cell input resistance (R_{in}) and sag. Sag_{sub} was calculated as (A-C)/A and sag_{fit} was calculated as (B-C)/B and expressed as a percentage. **C,D,** Western blots of lysates of primary cortical neurons (14 DIV) from WT and Tau35 mice, probed with antibodies to **(C)** phosphorylated tau (PHF-1), total tau, **(D)** HCN1, HCN2 or HCN3, and GAPDH. Quantification of the blots is shown in the graphs as mean ± SEM; *n*=5 independent experiments. Student *t* test, **P* < 0.05. **E**, Graphs show quantification of sag_{sub} and sag_{fit} voltage in WT and Tau35 mouse cortical neurons (11 to16 DIV). Graphs show mean ± SEM; *n*=5 (WT) and *n*=6 (Tau35) neurons. Student *t* test. **F**, I_K conductance in WT and Tau35 mouse hippocampal neurons (11 to 16 DIV). Average ± SEM values are shown in relation to different voltage steps. The example trace in the top insert, evoked by a depolarizing voltage step (30 ms) reveals the different voltage gated components evoked by depolarization. **G**, Graphs showing no effect of genotype on either the maximal conductance (Gmax) or the half-activation potential ($V_{1/2}$). Graphs show mean ± SEM, Student *t* test, *n*=12 to 16 neurons from 3 independent

experiments. **H**, AP-peak (amplitude) (mV) and AP-threshold (mV) of the plasma membrane and comparative action potential (AP) waveforms of WT and Tau35 neurons at 11 to 16 DIV. Graphs show mean ± SEM, *n*=8 to 11 neurons from 3 independent experiments. Student *t* test, $P > 0.05$. WT, wild-type; HCN, hyperpolarization-activated cyclic nucleotide-gated; GAPDH, glyceraldehyde 3-phosphate dehydrogenase; I_h, hyperpolarization-activated inward current; SEM, standard error of the mean.
